# A novel multisystem approach uncovers conserved mitochondrial complex I disease phenotypes and suppressors

**DOI:** 10.21203/rs.3.rs-10539896/v1

**Published:** 2026-08-11

**Authors:** Natascia Ventura, Lena Jentsch, Silvia Maglioni, Caleb Jerred, Tyler O’Hara, Eirini Mytilinaiou, Jimena Ruiz, Yulia Schaumkessel, Laura Schroeter, Jiwon Jung, Nikola Yotov, Johanna Hoffmann, Kai Kremers, Andrea Rossi, Andreas Reichert, Jennifer Watts, Konstantinos Palikaras, Suraiya Haroon, Alessandro Prigione

**Affiliations:** Heinrich Heine University Düsseldorf; University Hospital Duesseldorf. Heinrich Heine University; University Hospital Duesseldorf. Heinrich Heine University; Heinrich Heine University Düsseldorf; The Children’s Hospital of Philadelphia; National and Kapodistrian University of Athens; Washington State University; Heinrich Heine University Dusseldorf; University Hospital Duesseldorf. Heinrich Heine University; Leibniz Research Institute for Environmental Medicine; Leibniz Research Institute for Environmental Medicine; Leibniz Research Institute for Environmental Medicine; Leibniz Research Institute for Environmental Medicine; IUF-Leibniz Research Institute for Environmental Medicine; Institute of Biochemistry and Molecular Biology I, Medical Faculty and University Hospital Düsseldorf, Heinrich Heine University; Washington State University Pullman; National and Kapodistrian University of Athens; Children’s Hospital of Philadelphia; University Hospital Duesseldorf. Heinrich Heine University

**Keywords:** Mitochondria, Complex I deficiency, Leigh syndrome, Model organisms, NAM, C. elegans, Zebrafish, Brain organoids, Lutein, Vitamin B12 (cobalamin)

## Abstract

Mitochondriopathies are a large group of pathological conditions characterized by dysfunction of tissues and organs with high energy needs such as the brain, heart, and skeletal muscles, which highly rely on functional mitochondrial oxidative phosphorylation (OXPHOS). The vast majority of these disorders are ascribed to mutations in nuclear or mitochondrial encoded subunits of complex I (CI), the largest OXPHOS complex. In this study, we develop a multisystemic pipeline for CI-associated disease based on new approach methods (NAMs), which includes non-mammalian models, namely *C. elegans* and zebrafish, and mammalian cell-based models, such as neuroblastoma cells and induced pluripotent stem cell (iPSC)-derived neural progenitor cells (NPCs) and brain organoids. Systematic characterization of biochemical and neurobehavioral features in these models reveals obvious mitochondrial alterations with no major signs of redox imbalance possibly due compensatory or tissue-specific effects. Conversely, we identify specific pathogenetic features that are consistent across species and are rescued by lutein or vitamin B12, likely acting through non-cell-autonomous mechanisms converging on neurometabolic rewiring. Overall, our findings support the advantage of leveraging different *in vitro* and *in vivo* NAM systems with their unique and complementary disease endpoints to uncover critical pathological and quantifiable phenotypes exploitable to identify disease modifiers and suppressors.

## INTRODUCTION

Mitochondria-associated diseases (MD) are a vast group of pathologies characterized by dysfunction of tissues and organs relying highly on functional mitochondrial oxidative phosphorylation (OXPHOS), such as the brain, heart, and skeletal muscles^1^, thus often presenting with a broad spectrum of signs and symptoms, including neurodegeneration, cardiomyopathy, and metabolic abnormalities^2^. Most of these disorders are ascribed to mutations in nuclear or mitochondrial encoded subunits of complex I (CI) of the electron transport chain (ETC) or its regulatory proteins and are often associated with early-onset, fatal forms of pediatric diseases, such as Leigh syndrome^3,4^ (LS, OMIM #256000) also known as “infantile subacute necrotizing encephalomyelopathy”. Despite significant advances in our understanding of CI biology^5,6^, there are no cures for these devastating disorders and even symptomatic treatments remain limited. This lack of interventions is largely ascribed to the vast number of pathogenic mutations and variability of symptoms and to the restricted number of mammalian animal models that can accurately recapitulate the disease features. While disruption of CI structure is often described in CI-disease models, the severity of CI and OXPHOS dysfunction does not consistently correlate with it nor with a coherent pattern of disease signs and symptoms, and this is possibly due to cell-, tissue-, organ- and/or species-specific mitochondrial threshold effects and compensatory mechanisms^7^.

To gain insight into CI-associated disease pathogenesis and underlying molecular mechanisms, and accelerate the development of targeted therapeutic interventions, it is therefore crucial to develop disease models that appropriately recapitulate cellular pathology and clinical manifestations of CI deficiency. This is in line with current regulatory suggestions that promote new approach methods (NAMs) to model human diseases while reducing the reliance on mammalian animal use^8^. To this end, past efforts primarily leveraged unicellular *in vitro* systems (e.g. immortalized cell lines and patients’ fibroblasts), but establishment of 2D or even 3D cellular systems (e.g. induced pluripotent stem cells (iPSC)-derived organoids) as well as *in vivo* models (e.g. *Caenorhabditis elegans*, *Drosophila melanogaster*, and *Danio rerio*) recapitulating the human disorders is a growing effort in the field^3,7,9,10^. Each experimental system has its unique advantages and limitations with trade-offs between biological complexity, translational relevance and experimental accessibility^7^ and the choice of the disease model is usually guided by the specific research question and experimental objective. Limitations of currently available disease models for MD could, at least in part, be overcome by leveraging the complementary use of *in vivo* and *in vitro* NAM systems^11–14^.

With this work, to meet the urgent need of establishing reliable MD models exploitable to gain insight into pathomechanistic aspects and search for new therapeutic options, we developed a multisystemic pipeline for CI-associated disease primarily linked to pathogenic *NDUFS1* variants and characterized neuro-mitochondrial features of the different model systems. We specifically took advantage of neuroblastoma cells and iPSCs, engineered to carry the *NDUFS1* missense mutation c.691C > G (L231V) previously shown to cause LS^15,16^, to dig into biochemical and neurostructural features. SH-SY5Y cells are often used to study neuronal disorders^17^. For iPSC-based models, we focused on neural progenitor cells (NPC), as well as midbrain dopaminergic neurons (mDAN) and organoids (MBO), which, consistent with the specific defects occurring in the midbrain of LS patients, were previously shown to be dysfunctional in the context of LS models carrying other nuclear or mitochondrial variants in complex I, IV, and V^18–22^.

Cellular *in vitro* assays were complemented with neurobehavioral and metabolic endpoints *in vivo* in previously existing *C. elegans* and zebrafish models of CI deficiency^12,23^ and in newly generated *C. elegans* lines engineered to carry either one of two homozygous mutations found in LS patients in highly conserved residues, L231V^15^ and R408C^24^ (in *C. elegans* L234V and R412C respectively). Upon CI disruption we detected clear mitochondrial alterations, including defective respiration, isolated CI deficiency and reduced mitochondrial membrane potential, without obvious signs of redox imbalance. We also identified CI disease-associated features, such as altered cell/animal growth and defective neurometabolic parameters, which are consistent across species. Most notably, using key pathological endpoints we disclosed dietary supplements, namely lutein and vitamin B12, as protective interventions, most likely acting through converging neurometabolic rewiring via cell-non-autonomous and tissue-specific mechanisms. Overall, our findings support the advantage of coupling different NAM systems with their own unique and complementary features to identify critical pathological and quantifiable phenotypes exploitable for disease suppressor screening.

## MATERIALS AND METHODS

### Mammalian SH-SY5Y cell methods

#### Cell culture and differentiation

Neuroblastoma cells SH-SY5Y wildtype (WT) and knock in SH-SY5Y *NDUFS1* L231V cells were cultivated and maintained in growth medium (Dulbecco’s Modified Eagle medium (DMEM)/F12 GlutaMAX Supplement media (Gibco), 10% heat inactivated fetal calf serum (hiFCS) (Gibco), 1% Penicillin-Streptomycin) at standard conditions of 37 °C, 5% CO_2_ and 95% humidity. Cells were passaged at 80% confluency and cells of passage 3 – 15 were used for experiments. Less adherent neuron-like (N-type) cells of heterogenous cell population in *NDUFS1* L231V were enriched during the first three passages by knocking off N-type cells into Dulbecco’s Phosphate-Buffered Saline (PBS) and subsequent cultivation.

Neuronal differentiation of SH-SY5Y WT and *NDUFS1* L231V cells was induced by substituting growth medium with differentiation medium (DMEM/F12 GlutaMAX Supplement media, 1% hiFCS, 1% Penicillin-Streptomycin) supplemented with 10 μM All-trans retinoic acid (RA, 5 mM in 95% ethanol (EtOH), stored at 4°C for up to 6 weeks). Differentiation was started 24 h after seeding on extracellular matrix coated dishes and continued for 7 d whereby differentiation media was exchanged every 3 d. Differentiated cells from passage 4 – 10 were used for experiments. Protocol was adapted from Shipley, Mangold and Szpara^25^.

#### Cell Proliferation

The growth capacity and doubling time of SH-SY5Y WT and *NDUFS1* L231V were determined by seeding 8 × 10^5 cells of SH-SY5Y WT and *NDUFS1* L231V into 6-well plates and collecting and counting them after specified timepoints of 48 h and 96 h using Neubauer Cell Chamber. The doubling time of cells was calculated according to equation in Sherley, Stadler and Stadler^26^.

#### Mitochondrial Respirometry

Respirometry experiments in SH-SY5Y WT and *NDUFS1* L231V cells were performed using Seahorse XFe96 Analyzer (Agilent). SH-SY5Y WT and *NDUFS1* L231V cells were seeded at density of 2.5 × 10^4 and 3 × 10^4 cells/well respectively in Seahorse XFe96 well plates and incubated in growth media for 48 h. Seahorse Mito Stress Test was performed as according to manufacturer’s instruction. Before conduction of the assay media of cells was replaced by supplemented Seahorse assay media (10 mM glucose, 2 mM glutamine, 1 mM sodium pyruvate, all Agilent) and incubated for 30 min without CO_2_. The mitochondrial Oxygen Consumption Rate (OCR) was measured under basal condition followed by the sequential injection of oligomycin (1 μM), FCCP (1 μM) and rotenone/antimycin A (0.5 μM). To assess individual mitochondrial complex activity, SH-SY5Y WT and *NDUFS1* L231V cells were seeded at density of 2.5 × 10^4 and 3 × 10^4 cells/well respectively in Seahorse XFe96 well plates and incubated in growth media for 48 h. Prior to running the assay, the media was exchanged to assay media (0.11 M Mannitol, 70 mM Sucrose, 10 mM KH_2_PO_4_ 10, 5 mM MgCl_2_, 2 mM HEPES, 1 mM EGTA, 0.2% fatty acid free bovine serum albumin (BSA)) complemented with substrates 1mM sodium pyruvate, 1 mM malate and 4 mM ADP. To permeabilize cells XF Plasma Membrane Permeabilizer (Agilent) was added to assay media (1:1000 dilution). Mitochondrial OCR is measured under supply of mitochondrial CI substrates pyruvate and malate and during sequential injection of 2 μM rotenone to block CI, the CII substrate 10 mM succinate, 2 μM antimycin A to block CIII and 10 mM/200 μM ascorbate/TMPD as substrate for CIV. All OCR measurements were normalized on cell number using 4 μg/mL Hoechst dye in DPBS after running the assays. All data was analyzed using Wave Software, Microsoft Excel and GraphPAD Prism 11. Complex related respiration was calculated using following formulas adapted from de Mey et al.^27^:

CI-related respiration=(basal OCR)−(Rotenone OCR)

CII/CIII-related respiration=(Succinate OCR)−(Antimycin A OCR)

CIV-related respiration=(Ascorbate/TMPD OCR)

#### Clear Native PAGE and CI in-gel activity

Mitochondria were isolated by mechanical disruption of cells with a 20 G canula followed by sequential centrifugation. Mitochondria were resuspended in BSA-free lysis buffer and protein was quantified using Bicinchoninic acid (BCA) assay (Thermo Scientific). For Clear Native-Polyacrylamide Gel Electrophoresis (CN-PAGE) 100 μg of mitochondria are solubilized in solubilization buffer (50 mM NaCl, 2 mM 6-Aminohexanoic Acid, 50 mM Imidazoles/HCl, 1 mM EDTA, 2.5 g digitonin/g protein) for 45 min on ice. CN-PAGE samples were prepared with 0.01% Ponceau S and 5% glycerine and run on 3%−13% gradient gels to separate proteins at 150 V, 15 mA for 16 h. After electrophoresis gel is incubated in CI activity buffer (5 mM Tris/HCl, 0.1 g/L NADH, 2.5 g/L NBT) for 30 min at room temperature. Development of colored bands was detected using VILBER LOURMAT Fusion SL equipment (Peqlab).

#### Reactive oxygen species (ROS)

SH-SY5Y WT and *NDUFS1* L231V cells were seeded at density of 2 × 10^4 cells into 24-well plates and incubated for 24 h before treating cells with 1 μM rotenone (CI inihibitor) analyze the amount of general ROS a 30 min acute treatment with 1 mM H_2_O_2_ was included as a positive control. After collecting cells with accutase, they were stained with CM-H_2_DCFDA (Invitrogen #C6827, 10 μM in DPBS) for 30 min at 37°C, washed twice with DPBS and FITC signal was measured via flow cytometry. To analyze the generation of mitochondrial ROS a 30 min acute treatment with 12.5 μM antimycin A was performed as a positive control for the generation of superoxide. After collecting cells with accutase, they were stained with MitoSOX (Invitrogen #M36008, 2.5 μM in DPBS) for 30 min at 37 °C, washed twice with DPBS and PE signal was measured via flow cytometry. The flow cytometer BD Canto II was used, and dead cells were excluded by additional counterstaining with DAPI (1 μg/mL final concentration). The analysis of flow cytometry data was performed with FlowJo 10. and GraphPad Prism 11.

#### Lipid peroxidation

SH-SY5Y WT and *NDUFS1* L231V cells were seeded at density of 2 × 10^4 cells into 24-well plates and after incubation for 24 h before treating cells with 75 mM Glutamate for 24 h. Additionally cells were treated with 1 μM Liproxstatin-1 (Lip1) or co-incubated with 75 mM glutamate and 1 μM Lip1 to verify lipid peroxidation caused by glutamate. After collecting cells with accutase, cells were stained with BODIPY^™^ C11 (Invitrogen #D3861, 2 μM DPBS) for 30 min at 37 °C, washed twice with DPBS before measuring FITC and PE signal via flow cytometry. The flow cytometer BD Canto II was used, additional counterstaining with DAPI (1 μg/mL final concentration) was used to exclude dead cells. The analysis of flow cytometry data was performed with FlowJo 10 and GraphPad Prism 11.

#### Mitochondrial membrane potential (MMP)

SH-SY5Y WT and *NDUFS1* L231V cells were seeded at density of 2 × 10^4 cells into 24-well plates and incubated for 24 h. Cells were treated with 0.1 mM CCCP for 30 min as a positive control. Cells were collected with accutase and stained with JC-1 (Invitrogen #T3168, 10 μM in serum free DMEM/F12 media) for 30 min at 37 °C and washed twice with DPBS, before FITC and PE signal was measured via flow cytometry. The flow cytometer BD Canto II was used, the analysis of flow cytometry data was performed with FlowJo 10. and GraphPad Prism 11.

#### Neuronal Outgrowth

SH-SY5Y WT and *NDUFS1* L231V were seeded at density of 2.5 × 10^5 cells into 6-well plate and incubated and differentiated for 7 d as described before. After differentiation, cells were washed once with serum free DMEM/F12 and stained with Hoechst 33342 (5 μM in DMEM/F12) for 5 min at 37 °C before imaging cells in brightfield and fluorescence microscopy in DAPI channel at 20x magnification using Zeiss AxioObserver. Images were analyzed and neurite outgrowth was quantified using ImageJ software with NeuriteTracer Plugin^28^ and GraphPad Prism 11.

#### Cell viability

SH-SY5Y WT and *NDUFS1* L231V cells were seeded at density of 2.5 × 10^4 and 3 × 10^4 cells/well respectively into 96-well plates. After 24 h incubation cells were treated either with 200 – 500 μM Diethyl maleate (DEM), 100 – 500 μM H_2_O_2_ or 75 – 150 mM Glutamate and respective solvent control for 24 h. Supernatant was removed from cells and Resazurin working solution (Sigma Aldrich #199303, 20 μg/mL in serum free DMEM/F12) was added to each well, incubated for 3 h at 37°C, 5% CO_2_ and fluorescence was measured at Ex/Em 530 nm/590 nm at Tecan Infinite 200 PRO. Based on fluorescence data cell viability in % relative to solvent control was calculated and analyzed with GraphPad Prism 11.

#### Apoptosis

SH-SY5Y WT and *NDUFS1* L231V cells were seeded at density of 2 × 10^4 cells into 24-well plates and incubated for 24 h before treating with 300 μM DEM or 250 μM H_2_O_2_ for additional 24 h. Cells were collected with accutase, washed twice with cold DPBS and stained with Annexin V-FITC Apoptosis detection kit (Biovision, K101–100) according to manufacturer’s instruction using binding buffer with Annexin V-FITC conjugate and PI for 10 min at room temperature before measuring FITC and PE signal via flow cytometry. The flow cytometer BD Canto II was used, the analysis of flow cytometry data was performed with FlowJo 10. and GraphPad Prism 11.

#### Gene expression

SH-SY5Y WT and *NDUFS1* L231V cells were seeded at density of 2 × 10^6 cells into 10 cm plates, incubated for 72 h. For neuronal differentiation 8 × 10^5 cells were seeded into 10 cm plates and differentiated for 7 d as described before. Cells were collected and cell pellets were frozen at −20 °C before isolating RNA using RNeasy Plus Mini Kit (Qiagen #74134) according to manufacturer’s instruction. Lysis buffer was supplemented with 143 mM ß-ME and cells were lysed by vortexing for 30 sec. Isolated RNA was eluted into RNAse-free H_2_O. RNA quantity was spectrophotometrically determined using Thermo Fisher NanoDrop and 1 μg RNA was used for reverse transcription into cDNA using GoScript Reverse transcription mix and random primers (Promega #9PIA280) according to manufacturer’s instruction. Quantitative PCR (qPCR) was performed on 10 ng cDNA per reaction using iQ^™^ SYBR^®^ Green Supermix (Bio-Rad #170–8880) according to manufacturer’s instruction on Thermo Fisher Quant Studio 3. qPCR data was analyzed using QuantStudio Design and Analysis Software 1.3.1. and GraphPad Prism 11. Primer sequences used for qPCR are listed in **Supplementary Table 1**.

### Human iPSC-induced NPC, mDAN and MBO methods

#### Culture of iPSCs

iPSCs were cultured in StemMACS^™^ iPS Brew XF (Miltenyi) supplemented with Mycozap Plus-CL (Lonza) on tissue culture plates coated with 200 μg/mL Matrigel^®^ (Corning) at 37 °C with 5% CO_2_ and 5% O_2_. iPSCs were typically passaged every 4–5 d with EDTA (Invitrogen) and 10 μM ROCK inhibitor (Enzo Life Sciences) to promote cell survival.

#### Generation and culture of NPCs

NPCs were generated and cultured following the protocol described by Zink et al., 2021^29^. On day 0 of NPC generation, one well of 80% confluent iPSCs were detached with accutase, collected, and transferred to an uncoated 6 well plate with 5 mL of medium 1 containing KnockOut-DMEM (Gibco), 1x KnockOut-SR (Gibco), 2 mM glutamine (Gibco), 1x NEAA (Gibco), 1 mM pyruvate (Gibco), 1x Mycozap Plus-CL (Lonza), 3 μM CHIR (Caymen Chemical), 10 μM SB-431542 (MACS Miltenyi), 1 μM dorsomorphin (Sigma Aldrich), 500 nM purmorphamine (MACS Miltenyi), and 10 μM ROCK inhibitor (Enzo Life Sciences), allowing embryoid bodies (EBs) to form. On day 2, EBs were collected and 50% of the medium was changed to medium 2 containing “Basic Medium (sm-)”—1:1 Hams F12: Neurobasal (Gibco), with 0.5x N2 (Gibco), 0.5x B27 without vitamin A (Gibco), 1x Mycozap Plus-CL, and 2 mM glutamine—supplemented with 3 μM CHIR, 10 μM SB, 1 μM dorsomorphin, 500 nM purmorphamine. On day 3, EBs were collected and 100% of the medium was changed to Medium 2. On day 4, EBs were collected and 100% of the medium was changed to Medium 3 (sm+), containing sm- supplemented with 3 μM CHIR, 500 nM purmorphamine, and 150 μM vitamin C (Sigma Aldrich). On day 7 EBs were shredded by pipetting, transferred to tissue culture plates coated with 200 μg/mL Matrigel^®^ with sm+ containing 10 μM ROCK inhibitor, and incubated at 37 °C with 5 % CO_2_. NPCs were then cultured in sm+ and passaged until homogeneous. NPCs were typically passaged every 7 d with accutase (Sigma Aldrich).

#### Early branching in NPCs

NPCs were split with accutase, counted with trypan blue (Sigma Aldrich), and seeded to an f-bottom 96 well plate (Greiner) with 10,000 cells per well. Cells were cultured for 6 d to allow for outgrowth of branches. Cells were then live-stained with 1:10,000 Hoechst 33342 (Life Technologies) for 30 minutes and then imaged using the Zeiss Axio Observer microscope at 20x magnification with phase contrast and Hoechst filters. For each image set, CellProfiler (Version 4.2.5) was used to count the nuclei present in the Hoechst image and the number of branches was manually counted from the phase contrast image. Branches and nuclei were added across 4 image fields per well and total branches were divided by total nuclei to obtain final branches / nucleus count. 5 wells per condition were imaged.

#### MMP in NPCs

NPCs were split with accutase, counted with trypan blue, and seeded to an f-bottom 96 well plate (Greiner) with 50,000 cells per well. The next day, NPCs were stained for live imaging with TMRM (10 nM) (Invitrogen) and Hoechst 33342 (1:10,000) (Life Technologies) for 30 minutes. Negative controls were treated with 5 μM FCCP (Sigma Aldrich) and 5 μM antimycin A (Sigma Aldrich) to deplete MMP. Plates were then imaged with the Operetta CLS^™^ (Revvity) at 20x magnification. Images were processed with CellProfiler (Version 4.2.5) to quantify integrated TMRM intensity as described in by Zink et al., 2022^30^.

#### Generation of mDANs

mDANs were generated from NPCs using small molecules as described in Reinhardt et al., 2013^31^. Basal medium was prepared with 1:1 Hams F12:Neurobasal, with 0.5x N2, 0.5x B27 + vitamin A (Gibco), 1x MycoZap Plus-CL, and 2 mM glutamine. On days 0 – 7 mDANs were cultured in differentiation medium dsm+ containing basal medium supplemented with 100 ng/mL FGF8-a (StemCell Technologies), 1 μM purmorphamine, and 200 μM vitamin C. On days 8 – 9 mDANs were cultured in differentiation medium dsm2+ containing basal medium supplemented with 10 ng/mL BDNF (MACS Miltenyi), 10 ng/mL GDNF (R & D Systems), 1 ng/mL TGF-3β (StemCell Technologies), 200 μM vitamin C, 500 μM db c-AMP (StemCell Technologies), and 500 nM purmorphamine. From Day 10 on, mDANs were cultured in maturation medium dmsm+ containing basal medium supplemented with 10 ng/mL BDNF, 10 ng/mL GDNF, 1 ng/mL TGF-3β, 200 μM vitamin C, and 500 μM db c-AMP.

#### Microelectrode array recordings in mDANs

To measure electrical spiking activity, mDANs were seeded to 3Brain high density-microelectrode array (HD-MEA) chips. Chips were coated with poly-L-ornithine (Sigma Aldrich) and laminin (Sigma Aldrich). 6-week-old mDANs were singularized with accutase, counted with trypan blue, and 70,000 cells were seeded to the recording area of the chip with CEPT cocktail kit (Tocris Bioscience) to promote cell survival. Culture media was progressively changed to BrainPhys^™^ Neuronal Medium (StemCell Technologies), which was supplemented with small molecules in equal concentrations as dmsm+ medium, to encourage electrical activity. Recordings were taken for 5 min with BrainWave5 software. Active units were identified using BrainWave5 neuronal culture spike identification and spike sorting preset parameters. To analyze mean firing rate, the top 1% most active electrodes of the chip were selected and their mean firing rates were averaged.

#### MBOs Generation

MBOs were generated based on the protocol from Renner et al., 2021^32^. NPCs were split with accutase for 5 min and counted with trypan blue. 9,000 NPCs were seeded in 150 μL sm+ into a U-bottom, ultra-low attachment, 96 well plate (Corning). On day 2, media was changed to patterning medium (PM) containing basal medium, supplemented with 1 ng/mL BDNF, 1 ng/mL GDNF, and 500 nM purmorphamine. On day 6, medium was changed to maturation medium (MM) containing basal medium supplemented with 2 ng/mL BDNF, 2 ng/mL GDNF, 1 ng/mL TGF-3β, 100 μM db c-AMP, and 5 ng/mL activin A (StemCell Technologies). All subsequent feedings were performed 3 times a week with MM without activin A.

#### MBOs growth measurement

For each size measurement, 10 MBOs were imaged using the Nikon Eclipse Ni-Ts2 microscope. MBO area was then measured in FIJI (version 2.14.0/1.54f) using the ellipse tool. The mean organoid size for each cell line was calculated on day 1 and then subtracted from subsequent measurements to determine growth from day 1.

#### Microelectrode array recordings in MBOs

MBO electrical spiking was measured using the 3Brain HD-MEA. Week 13 MBOs were cultured in BrainPhys^™^ Neuronal Medium with small molecules in equal concentrations to MM for 7 d prior to recording. To record activity under epileptic-like conditions, MBOs were transferred to medium containing 100 μM 4-AP (Sigma Aldrich) for 1 minute before being transferred to the 3Brain HD-MEA chip prewarmed to 36.5 °C. Media was then aspirated from the chip, leaving a thin layer over the recording area, and spiking activity was recorded for 3 min with BrainWave5 software. Spikes were identified using BrainWave5 brain organoid presets. Mean firing rate was then calculated over time by averaging all electrodes from the organoid area.

#### Immunocytochemistry in NPCs, mDANs and MBOs

NPCs and mDANs were seeded to coverslips coated with 200 or 400 μg/mL Matrigel^®^ respectively. NPCs, mDANs, and MBOs were fixed with 4% PFA (Thermo Fisher) for 15 min. Prior to staining, samples were permeabilized with 0.3% Triton-X (BioRad) in PBS for 10 min and subsequently blocked with blocking solution, 10% Donkey serum (Sigma Aldrich) and 0.05% NaN_3_ serum (Sigma Aldrich) in PBS, for 1 hour. Primary antibodies were diluted in blocking solution and incubated on cells for 1 h at room temperature or overnight at 4 °C. Primary antibodies were then washed from the samples and secondary antibodies were prepared in the same manner. All antibodies used are listed in **Supplementary Table 2**. Coverslips were then mounted to slides with ProLong Glass Antifade Mountant (Invitrogen). Coverslips and MBOs were imaged with the Zeiss Axio Observer microscope.

### Protein expression analysis in SH-SY5Y cells and NPCs

#### Immunostaining and FACS analysis

SH-SY5Y WT and *NDUFS1* L231V cells were washed once with DPBS and fixed with 2% paraformaldehyde in DPBS for 15 min at room temperature and consecutively were permeabilized with 0.7% Tween-20 in DPBS for 15 min at room temperature. After washing cells one with PBS, 3 × 10^5 cells were stained in 100 μL of primary antibody in 1% BSA/0.5% Tween-20 in PBS over night at 4 °C. After washing cells with DPBS they were stained with 100 μL secondary antibody in 1% BSA/0.5% Tween-20 in DPBS for 30 min at room temperature. Cells were washed twice with DPBS before measuring FITC signal via flow cytometry. The flow cytometer BD Canto II was used, the analysis of flow cytometry data was performed with FlowJo 10. and GraphPad Prism 11. Antibodies used are listed in **Supplementary Table 2**.

#### SDS PAGE and Western blotting

SH-SY5Y WT and *NDUFS1* L231V cells were washed twice with DPBS and scraped in RIPA buffer on ice for three minutes. Whole cell protein of hiPSC derived NPCs was extracted from thawed cell pellets which were resuspended in RIPA buffer and incubated on ice for 3 min, while vortexing every minute. Whole cell lysates were centrifuged to remove cell debris. Protein concentration of whole protein lysate was determined using BCA assay. SDS PAGE samples containing 30 μg protein were prepared with Laemmli buffer, DTT and heated to 95°C for 5 min. SDS PAGES were run on 12% gels and subsequently transferred on PVDF membranes. After blocking with 5% milk in TBS-T the membranes were stained with primary antibodies over night at 4 °C. Subsequently membranes were washed and stained with secondary antibodies conjugated to horseradish peroxidase (HRP) for 1 h at room temperature. The chemiluminescence signals were imaged using ECL reagent (Abcam) and VWR Imager CHEMI Premium (Avantor). Antibodies used are listed in **Supplementary Table 3.**

#### Blue Native PAGE and Western blotting

Mitochondria were isolated by mechanical disruption of cells with a 20 G canula followed by sequential centrifugation. Mitochondria were resuspended in BSA-free lysis buffer and protein was quantified using BCA assay. For Blue Native PAGE (BN-PAGE) 100 μg of mitochondria are solubilised in solubilization buffer (50 mM NaCl, 2 mM 6-Aminohexanoic Acid, 50 mM Imidazoles/HCl, 1 mM EDTA, 2.5 g digitonin/g protein) for 45 min on ice. BN-PAGE samples were prepared with Coomassie (8g dye/g detergent) and glycerine (50% final concentration) and run on 3%−13% gradient gels to separate proteins at 150 V, 15 mA for 16h before transferring proteins on PVDF membranes. Subsequently the membranes were blocked in 5% milk in TBS-T for 2 h and stained with primary antibodies over night at 4 °C. Subsequently membranes were washed and stained with secondary antibodies conjugated to HRP for 1 h at room temperature. The chemiluminescence signals were imaged using Pierce ECL reagent (Thermo Scientific) and VILBER LOURMAT Fusion SL equipment (Peqlab). Antibodies used are listed in **Supplementary Table 3**.

### Nematode methods

#### C. elegans strains and maintenance

We employed standard nematode culture conditions^12^. All strains were maintained at 20 °C on Nematode Growth Media agar supplemented with *Escherichia coli* (OP50 or transformed HT115), unless otherwise indicated. All experiments were performed on young adult hermaphrodites unless otherwise indicated. Strains used in this work are listed in **Table 1**. New *C. elegans* strains were created via crossing according to^33^. GFP or DsRed positivity was ensured via fluorescent microscopy (Leica; MZ10 F modular stereo microscope and Invitrogen by Thermo Fisher Scientific; EVOS M7000 Imaging System) and PCR followed by restriction digestion (FastDigest Bsp119I, Thermo Scientific, BstBI and SpeI-HF^®^, NEB) was used to identify mutants carrying the point mutations R412C and L234V. Primers used as follow: for *nuo-5* R412C*(syb4409)* Forward Primer 5’- GATCGGAGATCAGTCGCGTT-3, Reverse Primer 5’-CTTTCCCCGATGCACACCT-3 and Forward Primer 5’- AGAATGGATCGGAGATCAGT-3, Reverse Primer 5’-GTCCCTGATAGACCACAAAT-3. For *nuo-5*L234V*(syb4395)* Forward Primer 5’ TGCTATCACGACCGTCTCTC-3, Reverse Primer 5’- ttggtgctccacaaaacata-3.

#### RNAi feeding

The following dsRNA transformed bacteria for feeding were derived from the Ahringer C. elegans RNAi library^34^: nuo-1 (C09H10.3), T20H4.5, F53F4.10 and nuo-5 (Y45G12B.1). All dsRNA bacterial clones (sequence validated), were grown to at least 2OD and then diluted to a concentration of 0.9 OD with empty vector (pL4440) expressing bacteria.

#### Life- and health-span

Survival analysis started from hatching and was carried out at 20 °C. At least 60 animals per condition were scored as dead or alive and transferred every day on fresh plates during the fertile period, and then every other day or every 3 d until death. Worms were considered dead when they stopped pharyngeal pumping and no longer responded to touch. Worms that died because of internal bagging, desiccation due to crawling on the edge of the plates, or gonad extrusion were scored as censored. These animals were included in lifespan analyses up to the point of censorship and were weighted by half in the statistical analysis. We calculated mean lifespan, standard deviation of the mean, and P value (Mantel-Cox regression analysis) from Kaplan-Meier survival curves of pooled population of animals coming from at least two independent replicas. For statistical analysis we used the Online Application for Survival analysis OASIS 2^35^.

#### Quantification of fertility

L4 hermaphrodites were individually cloned onto small (3 cm) NGM plates spotted with 50 μL of OP50 bacteria at 20 °C. The hermaphrodites were transferred to fresh plates at 24 h intervals. Hatched progeny was counted after 48 h to facilitate the count of the larvae. The brood size of each animal was calculated as the sum of total progeny produced during the entire fertile period.

#### Motility

##### Manual trashing assay.

To further define neuro-muscular differences on an individual level, we analyzed the swimming behavior (also known as thrashing)^36^ of *nuo-5* animals. Age-synchronized worms were transferred to a fresh unseeded plate at room temperature to remove leftover bacteria; then individual worms were transferred to an unseeded small NGM plate filled with 1 mL of S-Basal buffer. Worms were allowed to acclimatize for 5 min, and the number of completed thrashes per minute was counted using a hand counter. This was performed on 15 individual worms per replicate in 2 or 3 independent biological trials.

##### Automated tracking activity.

An automated assay was performed using the WMicrotracker ARENA (Phylumtech S.A.), which can detect the movement of the nematodes through the scattering of infrared (IR) light, captured by a built-in camera^37^. The software allows for multiple different parameters to be adjusted to the specifics of the experiment – duration of the recording, number of wells used for the experiment, temperature and blue light illumination to stimulate activity in the nematodes. The worms were kept on plates containing OP50 bacteria at 25 °C from egglay until the first day of adulthood was reached. A 24-well plate was prepared with 1 mL of S-Basal in each well. 25 animals of each strain were manually picked and placed in duplicate in different wells. The plate was then gently tapped a few times to stimulate the activity and was then transferred into the WMicrotracker ARENA. The settings for the experiment were set as follows: The internal temperature was set to 25 °C to mimic the conditions under which the animals were grown prior to the experiment; the duration was set to 4 h with blue light pulses being activated for one minute every five minutes. The nematodes react to the blue light pulse and that counteracts any effects of fatigue due to the duration of the experiment. The data were exported at 15 min intervals.

#### Synaptic activity

##### Aldicarb-induced paralysis.

The assay was carried out following protocols previously described by others^38^. Briefly, the animals were all L2/L3 larvae on one day of the assay; they were grown on RNAi plates before the beginning of the assay when 25/30 worms were transferred on a small spot (4 μL) of OP50 *E. coli* in the center of the agar plate. The small spot concentrates the animals into a small single field of view, thus making it easier for careful examination of all animals on a plate. We used NGM plates containing a final concentration of 1 mM final aldicarb (Sigma-Aldrich, 33386) concentration in the NGM. After placing the animals on to an aldicarb plate they were left at RT. Every 30 min, for a total of 5.5 h, the worms were assayed for paralysis. We define a worm as paralyzed by the absence of movement when prodded three times with a platinum wire on the head and tail.

#### DEM resistance

DEM works by conjugating the cellular antioxidant glutathione (GSH), stopping the neutralization of ROS and increasing oxidative stress. DEM plates were prepared analogous to the aldicarb plates. For this, a 4 M DEM (Sigma-Aldrich D97703) stock solution was prepared by dissolving the DEM in DMSO. It was added to the liquid NGM to a final concentration of 20 mM (0.5% DMSO) or 40 mM (1% DMSO). Plates were then spotted with 100 μL concentrated OP50 bacteria and left to dry next to an open flame for at least 2 h. To concentrate bacteria, 2 mL of the overnight growth were centrifuged at 4°C and 1000 rpm for 10 minutes, the supernatant was removed and 100 μL S-Basal/condition was added^39^. For the experiment 25–30 age synchronized worms per condition were transferred onto the plates and checked at different time points (0, 4, 8 24, 26, 28, 30 and 32 h). Worms that left the plate were removed from the analysis (censored). Worms were considered dead if they did not respond after repeatedly being prodded on the head and tail with a platinum pick.

#### Juglone resistance

Juglone is a quinone naturally occurring in plants of the Juglandaceae (Walnut) family and works by reducing molecular oxygen to form free ROS, increasing oxidative stress. Juglone plates were prepared analogous to the aldicarb and DEM plates. For this, a stock solution of 50 mM juglone (Sigma-Aldrich 420120) dissolved in DMSO was prepared and added to the liquid NGM for a final concentration of 200 μM. Plates were spotted with 10 μL OP50 and left to dry next to an open flame for at least 2 h^40^. For the experiment 20–25 age synchronized worms per condition were transferred onto the plates and checked every 60 min for a total of 6 h. Worms that left the plate were removed from the analysis (censored). Worms were considered paralyzed if they did not respond after repeatedly being prodded on the head and tail with a platinum pick.

#### Reactive oxygen species

The quantification was performed either in a solid or liquid setting using MitoSOX Red (Thermo Fisher M36008).

##### Solid Media:

Synchronous L3 stage worm populations of each strain were grown on NGM plates spread with bacteria and 10 μM MitoSOX Red. After 24 h incubation, nematodes were transferred to fresh NGM plates for one hour to clear their guts of residual dye.

##### Liquid Media:

The incubation with MitoSOX was carried out as described by Schröter et al., 2024^17^. Briefly, worms were synchronized via egg laying and collected at the L4 state using S-Medium. Once the worms were washed three times with S-Medium, ~15 worms were added into each well of a 96-well plate (total volume 100 μL/well), in a mix containing 15 μL of BSA and 25 μL OP50. MitoSOX was added to the wells of 96-well plate (at a final concentration of 10 μM) and the plate incubated in the dark at 20 °C for 24 h. As a positive control Paraquat (4mM) was used 2 h prior to removing the worms from the MitoSOX.

For imaging, in both settings, worms were added to a microscope slide and paralyzed with 15 mM Levamisole. Images were acquired immediately with a fluorescence microscope at 40x magnification and a DsRed filter and analyzed via ImageJ (http://rsbweb.nih.gov/ij/), drawing a circle over the terminal pharyngeal bulb to define the ROI.

#### Lysosome-related organelles (LROs) quantification

NileRed (Sigma-Aldrich 72485) was dissolved in sterile S-Basal and spread out on medium plates with 7 mL NGM and 200 μL bacteria to a final concentration of 50 nM. The plates were dried in the dark at room temperature and then stored at 4 °C protected from light. The worms were synchronized by egg-lay. The larvae were then grown in the dark and collected via washing with S-Basal three times, once they reached the first day of adulthood. To measure the fluorescence worms were added onto a microscope slide, paralyzed via NaN_3_, and covered with a slide. Images were taken through the DsRed channel of a Zeiss Axio Imager M1 microscope (Carl Zeiss, Inc., Cologne, Germany) at 10x magnification with an excitation wavelength of 545 nm and an emission wavelength of 572 nm for 250 ms. Images were analyzed via ImageJ, drawing a circle posterior to the terminal pharyngeal bulb and using the animals’ body walls as borders to define ROIs.

#### Lipid peroxidation

BODIPY^™^ 581/591 C11 can be used to quantify ROS in membranes and cells, detecting damages via lipid peroxidation. Worms were synchronized on OP50 plates until they reached the first day of adulthood. For the positive control, wildtype worms were placed on 20 mM DEM plates for 3 h, inducing additional damages via increased ROS levels. They were then collected by washing them three times with M9 and stained using a BODIPY-C11 (Thermo Fisher Scientific D3861) 10 μM solution dissolved in M9. Worms were then placed on a microscope slide and paralyzed via 15 mM Levamisole. Images were immediately taken with fluorescence microscope at 10x magnification for oxidized BODIPY-C11 at 498 nm for excitation and 526 nm for emission for 250 ms and for reduced BODIPY-C11 at 577 nm for excitation and 603 nm for emission for 200 ms. The images were analyzed via ImageJ with the ROIs drawn analogous to the NileRed treatment.

#### DGLA-and B12 supplementation

Plates containing fatty acid sodium salt (DGLA) were prepared according to Deline, Vrablik and Watts^41^. Briefly, fatty acid solution was prepared to a final concentration of 100 mM with purified water. The working stock was purged with argon or nitrogen to prevents oxidation. Working stock was stored in the dark until use and made fresh each day it is needed to ensure proper potency. For B12 a 500 nM working stock was prepared in purified water and filter sterilized. Fatty acid working stock and/or the 500 nM vitamin B12 working stock into were poured in the liquid NGM for the final concentrations desired. When adding DGLA, stirring time was used to purge the DGLA working stock with argon or nitrogen. After the agar was solidified, the plates were covered and stored at room temperature in a well-vented drawer to be protected from light oxidation. Plates were seeded with *E. coli* OP50 1–2 d after drying and incubated in a dark environment. Nematodes were synchronized by treating gravid hermaphrodites with alkaline hypochlorite solution (for a 10 mL solution: 0.5 mL of 5M NaOH, 2.5 mL of household bleach, and 7 mL H_2_O). Eggs were pelleted by low speed centrifugation, washed three times in M9 buffer (3 g KH_2_PO_4_, 6 g Na_2_HPO_4_, 5 g NaCl, 1 mL 1 M MgSO_4_, H_2_O to 1L. Add MgSO_4_ after autoclaving), resuspend in a 15 mL tube to a final volume of 4–5 mL of M9 buffer and rock on a shaker overnight. L1 larvae were added to the plates supplemented with 0.3 mM DGLA (20:3n-6) 2 d after seeding with bacteria. Worms were incubated at 20 °C for 3 d or until they reached the adult stage.

#### Scoring Germ Cell Destruction

Adult worms were scored for sterility using a dissecting microscope with high enough magnification to visualize embryos developing in the uterus. Germ cell loss appears as a clear uterus void of eggs.

#### Quantification of gene expression via transgenic reporters

Nematodes were placed in a 14 μL S-Basal drop on a microscope glass slide, anesthetized with 20 mM Levamisole, covered with a cover slide and immediately imaged. Pictures were acquired with an Imager2 Zeiss fluorescence microscope, 10x magnification and ZEN 2 (blue edition) software. In each experiment a minimum of 15–20 animals per condition were used. The GFP signal was quantified in the different strains as described before^12^. Briefly, in CL2166 strain the GFP intensity of the total body was quantified, for the LX929 the intensity of the second proximal head neuron was quantified. With the NV55 and vjIs105 strains the intensity of the fluorescence was quantified in the ventral nerve cord (VNC), specifically, a rectangular area was designed in the posterior half of the nematode, between the vulva and the tail, and the GFP intensity was quantified in the selected areas.

#### Quantification of Mitophagy

To monitor neuronal mitophagy, we used transgenic *C. elegans* strains expressing *unc-119p::TOMM-20::Rosella* in wild-type, *nuo-5(syb4409)*, and *nuo-5(syb4395)* backgrounds. This design allows for pan-neuronal expression of the Rosella biosensor, a dual fluorescent reporter that expresses pH-sensitive GFP and pH-insensitive DsRed, fused to a mitochondria-targeting sequence. Mitophagy was assessed by calculating the ratio of GFP to DsRed fluorescence, based on the principle that while under basal conditions both fluorophores emit signal, in mitochondria engulfed by autophagosomes, GFP is quenched due to the acidic environment. Therefore, a decreased ratio of GFP to DsRed fluorescence reveals mitophagy induction, whereas an increased GFP/DsRed ratio is indicative of mitophagy blockage^42^. Gravid adults of the aforementioned strains were placed on NGM plates seeded with OP50 bacteria to lay eggs. OP50 was inactivated (UV, 15 min). Progeny were synchronized at the L4 stage and transferred to freshly seeded plates. The following day, the young adult nematodes (day 1) were immobilized in drops of 40 mM tetramisole hydrochloride (Sigma-Aldrich, T1512), and images were acquired using the EVOS M7000 Imaging System (Invitrogen by Thermo Fisher Scientific). Imaging was performed under identical conditions across all replicates, capturing both red and green fluorescence channels in the head region using 10x magnification. Image quantification was performed using Fiji (ImageJ) software as previously described^42^, by splitting fluorescence channels and calculating the ratio of GFP to DsRed mean pixel intensity within the same region of interest (ROI). Statistical analysis was conducted using GraphPad Prism 9.5.

### Zebrafish methods

#### Strain

The previously published *ndufs2* mutant line (PMID: 40791373) was generated at the Children’s Hospital of Philadelphia (CHOP) Zebrafish Aquatics Core using CRISPR/Cas9 gene editing. Since the homozygous animals do not survive to adulthood, adult *ndufs2*^+/−^ zebrafish were mated to obtain healthy control (*ndufs2*^+/+, +/^) and homozygous mutant (KO) animals, which are identifiable by the lack of an inflated swim bladder and loss of righting reflex by 5-days post fertilization (dpf).

#### Zebrafish larvae preparation

Adult zebrafish were set pairwise in mating tanks in the afternoon, and resultant embryos were collected the following day at 0-dpf. Animals were maintained at 28 °C for the remainder of the experiments in embryonic water (E3: consisting of 5 mM NaCl, 0.17 mM KCl, 0.33 mM CaCl_2_, 0.33 mM MgSO_4_, and 10mM Tris-HCl (pH 7.2)). On 0 and 1-dpf, embryos were sorted to remove non-viable embryos. After sorting on 1-dpf, embryos were sanitized with 0.003% sodium hypochlorite in E3 and treated with pronase (Roche, Cat No. 10165921001, Indianapolis, IN) to remove chorion and promote uniform hatching the same day.

#### Imaging

To measure vitamin B12 (cyanocobalamin, Sigma-Aldrich, V2876) and lutein (TargetMol, T3S1858) efficacy on zebrafish larval morphology, the larvae were treated with solvent only or drug conditions on 1-dpf immediately following dechorionation without refreshment of media. The animals were imaged on either 5-dpf or 7-dpf using a light dissecting microscope (Olympus MVX10, Center Valley, PA), an Olympus DP73 camera, and Olympus CellSens^®^ imaging software. ImageJ was used to quantify the various parameters. To assess body length, the line tool was used to span the rostral-caudal axis, exclusive of the fin. In rare cases where the larva possessed a slight bend, the segmented line tool was utilized with a midpoint at the center of the swim bladder, or lack thereof. To measure liver area, the freehand tool was used to outline the crescent-shaped liver, distinguishable by its darkened appearance. These two metrics were measured and graphed in pixels. 10–14 individual larvae were used in each condition, and the condition was assessed 3 or 5 times.

#### Zebrafish larval swimming activity

Larvae were imaged using an automated imaging system (ZebraBox, Viewpoint Behavioral Technology, Montreal, Canada) and activity response data were collected using ZebraLab software. The larvae were continuously either untreated or treated with 0.05% DMSO, or 0.1 μM lutein starting from 1-dpf. At 5-dpf, the animals were transferred in 300 μL of their treatment media without the use of anesthetic to individual wells of a clear 96-well flat bottom polystyrene microplate (Corning, 3599). The larvae were first exposed to 100% light (or ~28000 lux) for 20 min to acclimate, followed by a cycle of 10 min in 0% light (dark) to trigger maximal movement and then 10 min in 100% light to assess basal movement. This light–dark cycle was repeated three additional times in a single experiment. In each of the 3–5 biological replicates, we tested 8 to 12 animals per condition. The average activity scores for each fish within each of the four cycles were again averaged to achieve the mean total activity per fish. Descriptive statistics were determined across all fish within a treatment per replicate.

## RESULTS

### Mammalian neuronal cells carrying *NDUFS1* L231V variant display decreased growth and differentiation capacity

To follow up on our previous work and further model CI-associated diseases we generated different genetic models carrying point mutations found in patients with LS using CRISPR/Cas9^43^. We introduced a homozygous missense mutation - L231V - in *NDUFS1* exon 8 in the neuroblastoma cell line SH-SY5Y and first confirmed that the mutation induced a significant decrease in NDUFS1 protein expression (Fig. 1 a, b). Somewhat surprisingly, qPCR analysis revealed no alterations in *NDUFS1* transcript expression, indicating that the L231V mutation impairs NDUFS1 protein expression at a post-transcriptional level (**Supplementary Fig. 1 a**). We confirmed that the reduced protein expression was not an effect specific to SH-SY5Y cells by introducing the *NDUFS1* L231V mutation in embryonic kidney HEK293T cells (**Supplementary Fig. 1 b, c**), which also showed significantly decreased protein expression compared to WT.

Given that the neuroblastoma cell line has the potential to differentiate into neuron-like cells^25,44^, we envisioned this model to be more relevant for modelling neurometabolic diseases caused by mitochondrial dysfunction and continued further experiments using only SH-SY5Y cells. A clear phenotypic characteristic of the SH-SY5Y *NDUFS1* mutant cells was their slow proliferation capacity, with a doubling time of 55.0 ± 2.4 h compared to 31.6 ± 3.9 h of the WT cells (Fig. 1 c). Moreover, representative brightfield images of adherent cells showed morphological differences between WT and the whole population of mutant cells (Fig. 1 d). Whilst SH-SY5Y WT cell line consists mainly of smaller, elongated N-type cells, the whole population of *NDUFS1* L231V cells consisted mainly of bigger, flatter S-type cells. Besides morphological differences the two cell types were also typically distinguished by their adherence properties, N-type cells being less strongly adherent than S-type cells. After enriching *NDUFS1* L231V line for N-type cells, the population morphologically resembled the WT cells.

The phenotypic appearance of the different cell types was confirmed by immunofluorescence analysis of the S-type specific protein Vimentin, which showed a significantly increased expression of Vimentin within the whole population of *NDUFS1* L231V cells in comparison to the WT cells and to the *NDUFS1* L231V N-type enriched cells, whose Vimentin content did not significantly differ from the protein expression in WT (Fig. 1 e). The level of NDUFS1 protein expression and the growth capacity of the mutant cell’s whole population (primarily S-type) were also significantly decreased compared to the WT and to the N-type enriched cells (Fig. 1 b, c). Thus, to rule out potential bias of using different cell types, further experiments were primarily performed in the N-type enriched *NDUFS1* L231V line (which we will simply refer to as *NDUFS1* L231V) and in WT (naturally N-type enriched) cells.

We then assessed cellular ability to differentiate into neuron-like cells. To this end, neuronal outgrowth was determined using brightfield microscopy and fluorescence microscopy of DAPI stained nuclei, to quantify the length of neurite extensions per cell. As expected, when differentiating SH-SY5Y cells into neuron-like cells using RA, the morphology of the cells changed and the dendritic extensions protruding from the cells’ bodies expanded, creating branches and connections between the cells (Fig. 2 f). The neuronal outgrowth was significantly increased after 7 d of differentiation in WT but not in *NDUFS1* L231V cells (Fig. 1 g). Consistent with this, the expression pattern of neuronal differentiation markers was altered in comparison to WT cells: the neuron specific enolase (NSE), an enzyme involved in glycolysis in neurons, ßIII Tubulin, a neuronal specific component of the cytoskeleton and neuroligin 3 (NLGN3), an adhesion protein regulating synaptic structure and function (Fig. 1 h), were significantly increased in WT cells after differentiation, but not in the mutant cells (Fig. 1 i). In agreement with this, the whole population of *NDUFS1* L231V also showed decreased neurite outgrowth and an altered expression of neuronal protein markers compared to WT cells upon 7 d of differentiation (**Supplementary Fig. 2 a-d**).

Given that we previously described altered synaptic gene expression upon CI deficiency across different model systems^12^ we further analyzed the transcript expression of proteins involved in synaptic formation and function like neuroligins (NLGN1–4) and neurexin 1 (NRX1) in WT and *NDUFS1* L231V cells. Our analysis revealed a significant downregulation of *NLGN2* and a slight downregulation of *NRX1* in *NDUFS1* L231V cells compared to WT cells in basal, undifferentiated conditions (**Supplementary Fig. 1 d**). After neuronal-like differentiation *NLGN1* was significantly upregulated in both WT and *NDUFS1* L231V cells, whereas *NRX1* was only upregulated in WT cells in comparison to undifferentiated cells. *NLGN2* and *NLGN3* were downregulated in WT and *NDUFS1* L231V cells, whereas the expression of the *NLGN4* and *GCH1*, a gene regulating the generation of neurotransmitter dopamine remained unchanged (**Supplementary Fig. 1 e**).

Overall, characterization of phenotypic features indicates that mammalian neuron-like cells carrying *NDUFS1* L231V variant display decreased growth and differentiation capacity.

### *NDUFS1* L231V mutated cells display mitochondrial alterations

Besides phenotypic characteristics, we assessed the impact of the *NDUFS1* L231V mutation on mitochondrial structure and function. Mitochondrial CI is composed of three structural modules (N-, Q- and P-) that are formed stepwise out of many nuclear and mitochondrial DNA-encoded protein subunits forming intermediate preassembly complexes during biogenesis. Initially, the P- and Q-module are generated separately and subsequently combine to form the P/Q preassembly complex, which then combines with the N-module to form the fully assembled functional holoenzyme. CI further associates with complex III (CIII) and complex IV (CIV) of the ETC to form higher-order supercomplexes, which influence electron transport efficiency and overall mitochondrial respiration capacity. Supercomplexes occur in multiple stoichiometric configurations, ranging from CIII_2_ + CIV at a size of 750 kDa up to larger supercomplex formed by CI_1_+CIII_2_+CIV, known as the respirasome, with a size of >2 MDa^45,46^. Consistent with other CI disease cellular models^47^, BN-PAGE followed by western blotting of OXPHOS complexes and individual CI subunits NDUFS4, NDUFS2 and NDUFB6 (belonging to the N-module, Q-module and P-module respectively), revealed the following alterations in mitochondrial CI structure and in the formation of supercomplexes in SH-SY5Y *NDUFS1* L231V cells (Fig. 2 a–c). Specifically, an OXPHOS antibody cocktail primarily revealed a band at 1000 kDa corresponding to the holo CI and a band at around 750 kDa size containing CV and supercomplex consisting of CIII dimer CIII_2_ and CIV often visible in BN-PAGEs and thus used as a molecular marker (Fig. 2 a). Band patterns for the N-module NDUFS4 subunit did not show differences between WT and mutant cells, implying that the amount of preassembly complexes, holo CI and supercomplexes containing a fully assembled N-module in CI is comparable between the two cell lines (Fig. 2 b). Instead, the distinct band pattern upon staining of Q-module NDUFS2 subunit, indicated that preassembly of CI complexes below 750 kDa as well as holo CI at 1000 kDa and the supercomplex and respirasome formation at sizes of >2 MDa are dramatically reduced in *NDUFS1* L231V cells compared to WT (Fig. 2 b). Accordingly, a decreased formation of supercomplexes at molecular weight below and above 1000 kDa as well as at the respirasome sizes of >2 MDa (revealed by the missing distinct band pattern above 1000 kDa), was also detected in the mutant cells when staining for P-module NDUFB6 subunit, whereas the preassembly complexes consisting of the Q/P-modules at a size of 750 kDa remained unchanged (Fig. 2 b).

Additionally, BN-PAGE and western blot membranes were also stained for SDHA (succinate dehydrogenase), a subunit contained in CII, and for UQCRC2 a subunit of CIII (Fig. 2 c). SDHA staining revealed protein bands corresponding to CII at approximately 240 kDa, which were overall similar in WT and *NDUFS1* L231V cells. When staining UQCRC2, distinct bands below 750 kDa showed the presence of CIII dimer CIII_2_, which is also associated with multiple supercomplexes, as identified by the distinct protein band pattern at sizes of >1000 kDa. Consistent with the observations from the NDUFS2 and NDUFB6 stained membranes, this UQCRC2 band pattern was decreased in *NDUFS1* L231V cells, indicating decreased formation of supercomplexes containing CIII. Although BN-PAGE analysis indicated potential defects in CI assembly and supercomplex formation – processes known to be essential for mitochondrial respiration – CN-PAGE In-gel CI activity assay, using NADH as a substrate and NBT as an electron acceptor to produce visible color signal, did not reveal alteration of CI activity in *NDUFS1* L231V cells in comparison to WT cells (**Supplementary Fig. 3 a**).

Therefore, we then characterized mitochondrial respiration using Seahorse XFe96 Analyzer (Fig. 2 d). We found that basal and ATP-linked respiration were significantly decreased (from 85.4 ± 28.6 pmol/min/Norm. Unit to 17.4 ± 3.9 pmol/min/Norm. Unit and 66.9 ± 21.9 pmol/min/Norm. Unit and 13.0 ± 2.0 pmol/min/Norm. Unit respectively) in *NDUFS1* L231V compared to WT cells (Fig. 2 e, f). Moreover, individual ETC complex activity further supported a defect in mitochondrial respiration (Fig. 2 g) and specifically revealed an isolated CI deficiency in *NDUFS1* L231V cells, which retained 76% CI-linked respiration compared to WT cells (Fig. 2 h). Previous analysis indicated a more severe isolated CI defect, with residual CI activity of 25% in patients’ muscle homogenate^15^, differences that can possibly be ascribed to cell (neurons vs muscle) or assay (CI-linked respiration/Seahorse vs CI-enzymatic activity/spectrophotometry)-specific effects. Furthermore, staining of WT and *NDUFS1* L231V cells with the MMP probe JC-1 followed by FACS analysis indicated a significant decrease of MMP in the mutant cells compared to WT (Fig. 2 i). Consistently, analysis of mitochondrial respiration of the entire population of *NDUFS1* L231V confirmed a strong decrease of basal and ATP-dependent respiration (**Supplementary Fig. 3 b-d**).

Defects in mitochondrial respiration could lead to increased ROS formation. Levels of hydroxyl radical within the cells under basal conditions as well as after treatment with pro-oxidants rotenone and peroxide were quantified with H_2_DCFDA staining followed by FACS analysis (Fig. 2 j). Although under basal conditions ROS levels seemed to be slightly increased in *NDUFS1* L231V cells, both WT and mutant cells reacted similarly towards treatment with stressors, displaying equal increase in hydroxyl radical production. We then assessed mitochondrial ROS by staining WT and *NDUFS1* L231V cells with the mitochondrial superoxide specific probe MitoSOX followed by FACS quantification and found that antimycin A-induced ROS production was significantly increased in both cell lines. Interestingly, *NDUFS1* L231V cells displayed a significantly lower increase of mitochondrial ROS than WT cells (Fig. 2 k).

To further evaluate the impact of the *NDUFS1* L231V mutation on cellular redox homeostasis, we examined cell viability following exposure towards different oxidative stressors. Mitochondrial CI disruption was shown to increase susceptibility to ferroptosis^48–50^ – a peroxide and iron-induced form of cell death driven by lipid peroxidation and inhibited by glutathione (GSH)-dependent reactions –, which can regulate both neuronal development and differentiation^51,52^. We thus specifically investigated cellular oxidative stress induced by peroxide or GSH depletion, the latter achieved either through the GSH-conjugating agent DEM or by glutamate (which prevents cellular uptake of cystine, the rate-limiting precursor for GSH synthesis). Contrary to our expectation, (resazurin based) cell viability assays indicated that *NDUFS1* L231V cells were only slightly more sensitive to peroxide but less sensitive to DEM and equally sensitive to glutamate compared to WT cells (Fig. 2 l). Annexin V-propidium iodide staining followed by FACS analysis revealed also a non-significant trend towards increased apoptosis of *NDUFS1* L231V cells after treatment with DEM or peroxide (**Supplementary Fig. 4 a-b**). Staining with BODIPY^™^ 581/591 C-11 probe was then performed to assess lipid peroxidation as a hallmark of ferroptosis, which revealed a non-significant increase in the mutant cell, reversed by ferroptosis inhibitor liproxstatin-1(Lip-1) (**Supplementary Fig. 4 c**). Moreover, no major changes in the expression of ferroptosis inhibitory genes^53–55^ were detected in the *NDUFS1* L231V cells before or after differentiation (**Supplementary Fig. 4 d-e**). Indeed, whilst qPCR analysis revealed downregulation of *SCD1* expression in *NDUFS1* L231V cells in comparison to WT cells, the expression of *GPX4*, *ACSL3* and *PPAR* alpha remain unchanged (**Supplementary Fig. 4 d**). Further differentiation for 7 d did not affect the expression of ferroptosis related genes in WT and *NDUFS1* L231V cells **(Supplementary Fig. 4 e**).

Data described so far indicate that the mammalian cell line harboring *NDUFS1* L231V displays clear defects in mitochondrial biochemistry and function, which likely account for its defective proliferation and differentiation ability, yet without overt alterations in cellular redox homeostasis and ability to handle oxidative stress.

### *NDUFS1* L231V iPSC-derived NPCs, mDANs, and MBOs exhibit mitochondrial and functional defects

To further assess pathological disease features in a more relevant human neuronal system, we generated iPSCs carrying the same *NDUFS1* pathogenetic variant (by introducing the *NDUFS1* mutation c.691C>G into healthy parental cell line IPS12–10). We compared this iPSC line to the respective unedited isogenic control line. *NDUFS1* mutant iPSC line displayed normal karyotype and genomic integrity as well as expected morphology, pluripotency capacity, and ability to differentiate into all three germ layers^16^. Neurodevelopmental and mitochondrial pathologies induced by the mutation were then investigated by differentiating the iPSCs into NPCs, followed by generation of mDANs and MBOs.

Mutant NPCs displayed typical neuronal precursor markers, such as PAX6, Nestin, SOX2 and phosphorylated-Vimentin (Fig. 3 a; **Supplementary Fig. 5 a**) but appeared to spontaneously differentiate in culture before neuronal induction as seen by premature neurite branch formation (Fig. 3 b–c). The expression level of NDUFS1 protein was significantly decreased in the mutant NPCs compared to the isogenic control NPCs (Fig. 3 d; **Supplementary Fig. 5 b**). The expression of the CI core subunit NDUFS2 and of the accessory subunit NDUFS4 were also significantly lower, while the expression of the CI accessory subunit NDUFB6, as well as CII and CIII subunits were not affected (Fig. 3 e; **Supplementary Fig. 5 b-d**). Mutant NPCs also showed a significant decrease in MMP compared to the isogenic control line (Fig. 3 f). Thus, consistent with the effect of *NDUFS1* mutation in the neuroblastoma cell line, mutant NPCs display obvious mitochondrial defects.

Differentiation of NPCs into mDANs revealed altered growth and morphology in *NDUFS1* L231V mutant mDANS, with reduced axonal branching formation, as indicated by severely decreased expression of the pan-axonal marker SMI312, yet expression of the dendritic marker (MAP2), the dopaminergic neuron marker tyrosine hydroxylase (TH) and astrocyte differentiation markers (S100β and GFAP) remained unaltered (Fig. 4 a; **Supplementary Fig. 6 a-b**). Despite normal expression of dendritic markers, *NDUFS1* mutant line showed poorly structured and disorganized neurites (Fig. 4 b). Consequently, mutant mDANs exhibited a significantly lower firing rate compared to control mDANs when tested via HD-MEA (Fig. 4 c; **Supplementary Fig. 6 c-d**). These activity defects appeared stronger in the early timepoints following neuronal seeding, while they tend to normalize after 30 d on the MEA chips (Fig. 4 c), possibly indicating that mutant *NDUFS1* neurons had a delayed maturation trajectory.

NPCs were also differentiated into MBOs which displayed typical expression of dendritic (MAP2) and axonal (SMI312) markers (**Supplementary Fig. 7 a**). While isogenic control MBOs consistently grew from day 1 to day 10, the growth of mutant MBOs was significantly delayed (Fig. 4 d–e). The action potentials of *NDUFS1* L231V MBOs reflected typical average waveforms similar to WT (**Supplementary Fig. 7 b**). However, the altered growth capacity of mutant MBOs was accompanied by an increased mean firing rate upon 4-Aminopyridine treatment, which was used to induce epileptic behavior (Fig. 4 f), consistent with epilepsy being a frequent symptom of LS patients^56^. The increased epileptic-like firing upon potassium channel blockade establishes a human *in vitro* correlate of epilepsy as a major clinical symptom, and represents a significant advance over existing LS iPSC-derived organoid models which characterized corticogenesis defects without MEA analysis^57^.

Overall, data from mutant *NDUFS1* L213V iPSC-derived neurons and midbrain organoids recapitulate mitochondrial disruption of CI holoenzyme and respiratory supercomplex assembly observed in the mutant neuroblastoma cells, and further support deficits in neuronal growth, differentiation and development ultimately impacting cell functionality.

### *C. elegans NDUFS1*/*nuo-5* mutants display altered development and neurobehaviors

Data described so far leveraging the cellular systems with different complexity and relevance for the human disease, clearly indicate *NDUFS1* homozygous mutation induces mitochondria and neuronal structural and functional defects *in vitro*. To investigate the effects of *NDUFS1* mutations *in vivo*, we then turned to *C. elegans*, a multicellular organism widely employed to study the physiopathology of the nervous system and to model human neurometabolic diseases, including mitochondriopathies^10,58^. We previously showed that *C. elegans NDUFS1/nuo-5* silenced animals display mitochondrial and synaptic deficits, the latter corrected by a dietary carotenoid^12,59^. Mitochondrial functions and the structure of the ETC are highly conserved between *C. elegans* and mammals and the *nuo-5* gene sequence displays a high degree of homology to human *NDUFS1*, with 73.1% similarity and 57.2% identity (**Supplementary Fig. 8 a**). We generated two *C. elegans* CI-associated disease models carrying orthologous point mutations *nuo-5(syb4395)*L234V and *nuo-5(syb4409)*R412C, previously identified in LS patients, L231V and R408C respectively^15,24^ (**Supplementary Fig. 8 b-c**), which we exploited for complementary *in vivo* phenotypic and behavioral studies.

Post-embryonic development was slightly affected in the L234V mutant and evidently more in the R412C mutants, which reached fertile stage about 12 h after the control worms (Fig. 5 a). Similarly, brood size and lysosome-related organelles (LROs) tended to be lower in the L234V worms while being significantly decreased in the R412C mutants (Fig. 5 b–c). In *C. elegans*, according to the mitochondrial threshold effect, phenotypic changes only appear once a certain degree of mitochondrial stress is achieved^39,60^. Development timing, fertility and LRO content of the R412C mutants resemble the typical phenotypes observed in other genetic or RNAi-mediated mitochondrial mutants^61–63^ and, accordingly, also its lifespan and health span (measured by spontaneous movement over time) were significantly extended compared to WT, while those of the L234V mutants were not affected (Fig. 5 d–e). These phenotypes align with the concept of mitohormesis providing resistance to stressors^39,64,65^. However, the resistance of the two mutants to oxidative stress (DEM-induced glutathione depletion and juglone-induced production of ROS) was not unambiguously affected compared to WT animals (Fig. 5 f–g). This is possibly ascribed to differential activation of compensatory pathways such as glutathione-S-transferase (**Supplementary Fig. 9 a**), which may account for buffering ROS production. Indeed, contrary to our expectations, we observed no major changes in systemic mitochondrial superoxide production or lipid peroxidation (**Supplementary Fig. 9 b-d**). Instead, further analysis of animals’ response to stress indicated that both *nuo-5* L234V and *nuo-5* R412C mutants are more sensitive than WT to dietary DGLA-induced ferroptosis (Fig. 5 h), suggesting a germline-specific sensitivity to stress.

Moreover, we found that nematodes carrying the R412C mutation (but not the L234V mutants) exhibit impaired neuronal mitophagy, as indicated by the Rosella reporter, with an increased GFP/DsRed ratio compared to WT animals. (Fig. 5 i, **Supplementary Fig. 9 e-f**). When we looked at mitochondrial number and morphology, we did not observe any difference with the control (**Supplementary Fig. 9 g-i**), but consistently with an altered mitophagy machinery, the size of the mitochondria in the R412C mutants was increased (**Supplementary Fig. 9 j**).

A powerful feature of the nematode system is the possibility to specifically assess neurobehavioral parameters *in vivo*. We first tested the motility of the two mutants through the thrashing assay and found that *nuo-5* R412C mutants display a significantly reduced number of body bends (Fig. 5 j). Interestingly, an automated multi-worm tracking assay recorded increased activity in these mutants (**Supplementary Fig. 9 k**). This apparent discrepancy might be ascribed to the different parameter monitored by the device (overall animals’ activity) which, together with the number of thrashes, may actually reveal a non-effective locomotor activity, reminiscent of an epileptic behavior - as also suggested by the MEA analysis in the above-described brain organoids. Further support for impaired neuromuscular activity is highlighted by altered synaptic activity, measured as increased sensitivity to aldicarb-induced paralysis, in the R412C mutants compared to WT (Fig. 5 k). Our previous findings identified *nlg-1*, the only *C. elegans* neuroligin (a neuronal adhesion protein necessary for synaptic structure and function), as a crucial player in mediating the synaptic deficit in the *nuo-5* RNAi-depleted animals^12^. Therefore, we crossed an *nlg-1* fluorescence reporter line with the R412C mutant and observed a statistically significant increase in NLG-1 protein expression compared to WT worms (Fig. 5 l). Similarly, we found that *unc-17*, a gene required in cholinergic neurons for loading acetylcholine into synaptic vesicles and previously found to be differentially regulated in *nuo-5* RNAi-mediated mutants^12^, was also significantly increased by the *nuo-5* R412C mutation (Fig. 5 l).

Overall, our new *C. elegans* strains display mutation-dependent pathological changes at the systemic level without overt evidence of oxidative stress. Their developmental and neurobehavioral phenotypes provide relevant readouts for the identification of disease suppressors.

### Lutein and vitamin B12 rescue neurometabolic deficits observed in *C. elegans* and *Danio rerio* CI mutants

In previous studies we screened different natural compounds and identified lutein, kahalalide F, isovitexin and EGCG for their ability to promote healthspan in WT animals via mitohormetic mechanism or in *NDUFS1/nuo-5* RNAi-depleted *C. elegans*^12,66^. Namely, we found lutein protects against neuroligin-mediated synaptic defects in *nuo-5*-deficient animals^12^. We subsequently tested the identified compounds for their ability to ameliorate defects in animals’ growth, motility, synaptic activity and synaptic genes expression (*nlg-1* and *unc-17*) in different complex I subunits depleted by RNAi (**Supplementary Fig. 10 a-f**; and *data not shown*). We confirmed that lutein suppressed these defects in *nuo-5* RNAi-treated animals (only for one generation compared to our previous findings) and found that only lutein and kahalalide F ameliorate both motility and synaptic activity in different RNAi-mediated CI mutants (**Supplementary Fig. 10 a-h**). Moreover, a combination of lutein and kahalalide F had a synergistic effect and significantly rescued motility and synaptic transmission defects in *NDUFV1*/*nuo-1* RNAi-mediated mutants (**Supplementary Fig. 10 j-l**).

Given the consistent beneficial effect of lutein in different mitochondrial CI disease models, we then tested its ability to suppress phenotypic and behavioral deficits observed in the newly generated *nuo-5* R412C genetic mutants. Consistent with the RNAi-depleted study, lutein significantly ameliorated the thrashing defect in R412C animals (Fig. 6 a) and rescued the aldicarb sensitivity back to WT levels (Fig. 6 b). The expression of synaptic genes, *unc-17* and *nlg-1* which was increased in the R412C mutants, was diminished by lutein, although it reached statistically significant effect only for *nlg-1* (Fig. 6 c–d).

While testing lutein’s effects in *nuo-5* (RNAi or genetic) depleted animals we noticed diet-dependent effects (**Supplementary Fig. 11 a-f**). Dietary components, including carotenoids, may influence neuronal physiopathology through the gut-brain axis across species^67–70^. The nutritional difference between *E. coli* OP50 and HT115 lies primarily in their carbohydrate content, metabolic profiles, and vitamin B12 content, which can be linked to mitochondrial health^71–73^. Moreover, B12 produced by gut bacteria has been shown to modulate synaptic activity in *C. elegans* impacting on cholinergic signaling^74^. Notably, we tested the effect of B12 supplementation and found it to rescue animals’ defects in growth, motility and synaptic activity as well as the DGLA-induced sterility in the *nuo-5* genetic mutants (Fig. 6 e–h), the latter also suppressed by choline supplementation (**Supplementary Fig. 11 g**).

Overall, characterization of the newly generated nematode lines indicates that CI disruption, while producing phenotypic changes typically associated with other mitochondrial mutants, it also induces neurodevelopmental alterations of relevance for human MD pathology, which are significantly rescued by nutritional interventions.

To corroborate our *in vivo C. elegans* findings with the dietary compounds, we asked whether lutein and B12 could also ameliorate defects in another multicellular model of CI-associated disease. A zebrafish model for CI deficiency (*ndufs2*−/−) has been recently developed recapitulate some relevant aspects of CI disease pathology. Specifically, *ndufs2−/−* larvae exhibited reduced growth, neuromuscular dysfunction and morphologic malformations including hepatomegaly (**Supplementary Fig. 12 a-e**) at 5 days post-fertilization (dpf), which could be rescued by folic acid treatment^75^. In support of an evolutionary conserved role of one-carbon metabolism in CI-disease pathology, we observed that also vitamin B12 significantly rescued the increased liver growth in the *ndufs2−/−* fish larvae (Fig. 7 a–b). Rescue effects on animals’ body length and liver pathology were also observed when treating *ndufs2−/−* zebrafish with lutein (Fig. 7 c–e).

In summary, we identified different neurometabolic pathological features associated with CI-deficiency and rescued by dietary interventions across species.

## Discussion

Mitochondrial CI deficiency represents one of the most prevalent causes of MD, frequently affecting tissues with high energetic demand such as the nervous system. Despite substantial advances in the molecular characterization of CI, the mechanisms linking primary CI defects and OXPHOS dysfunction to tissue-specific pathology and clinical manifestations remain incompletely understood, partly due to the limited availability of model systems that faithfully recapitulate the systemic nature of these disorders. This poses significant limitations in the development of effective therapeutic interventions, which to date remain unfortunately critically limited^7,76,77^. In this study, in line with current regulatory indications in favor of NAMs to study human diseases^8^, we established a multisystemic pipeline to investigate the consequences of NDUFS1-associated CI deficiency across *in vitro* (neuroblastoma cells and human iPSC-derived neuronal systems) and *in vivo* non-mammalian animal models (*C. elegans* and zebrafish). *NDUFS1* disruption consistently resulted in decreased NDUFS1 expression and defective mitochondrial CI assembly and function, accompanied by impaired cellular and animal growth and differentiation, as well as structural and functional neuronal deficits. Moreover, we identified neurometabolic and neurobehavioral alterations across species, with lutein and vitamin B12 as two dietary interventions rescuing key pathological features, suggesting the involvement of conserved mechanisms, possibly acting via the gut-brain axis. Our multisystemic, integrative approach enables the identification of conserved pathological features and provides a robust pipeline to explore both disease pathogenesis and potential therapeutic opportunities.

A central observation emerging from our study is the remarkable consistency of pathological features across models of increasing biological complexity. Despite intrinsic differences between experimental systems, *NDUFS1* disruption resulted in a shared spectrum of mitochondrial, metabolic, and neuronal alterations across species. At the cellular level, decreased expression of CI protein (transcript expression was not affected) was associated with defective CI assembly and supercomplex organization, resulting in isolated CI deficiency and reduced basal and ATP-dependent mitochondrial respiration and membrane potential, which are often observed in other mitochondriopathies ascribed to mutations in CI encoded subunits^3,7^. Mitochondrial defects were accompanied by impaired cellular proliferation (likely reflecting an adaptive response to metabolic stress) and neuronal differentiation. These alterations were recapitulated in human iPSC-derived systems, where mutant neural progenitors displayed premature differentiation and mitochondrial dysfunction as also observed in other MD models^78^, while derived neurons and organoids exhibited impaired neurite organization and altered electrophysiological activity. *In vivo* models further extended these findings, revealing defects in organismal development and neurobehaviors. Noteworthy, the convergence of dysregulated neuronal firing in brain organoids (increased 4-AP-induced firing) with the paradoxical locomotor behavior in *C. elegans* (increased tracking activity, reduced effective thrashing accompanied by aldicarb hypersensitivity), represents a novel important cross-species endophenotype reminiscent of an epileptic-like state, mirroring neurological manifestations frequently observed in patients with LS^79^.

Interestingly, as also described with other CI mutations in human or in *C. elegans*^3,80^, we observed a consistent differential severity across multiple *C. elegans* neurometabolic endpoints (developmental timing, brood size, motility, aldicarb sensitivity, lipid metabolism) induced by the two homozygous mutations, *nuo-5* L234V being less severe than *nuo-5* R412C. This variant-specific severity is notable because the corresponding human mutations, L231V and R408C, in highly conserved amino acids and gene regions, were originally identified in severe LS cases^15,24^, presenting with ~ 25% residual CI activity in muscle homogenate and leading to fatal early-life outcomes at 14 and 8 months respectively. The discrepancy between the severity of the phenotypes in *C. elegans* and in human may thus result from species (Bruno Hay Mele, *personal communication*) or context-specific structural consequences of the two mutations. In support of our findings, the L231V/L234V substitution, unlike the R408C/R412C, does not alter the amino acid polarity and may therefore exert a more limited impact on CI conformation. Consistently, the mammalian L231V mutant cells analyzed in this study retained substantial residual CI activity, although a direct comparison between the two variants in the same cellular or animal context will be required to formally test mutation-specific biochemical severity. Indeed, we also speculate the intrinsic genetic and environmental variability in human, which are absent in *C. elegans*, may modify disease penetrance and progression, preventing a direct causal association between primary genetic defect and its biochemical and neuronal consequences.

Strikingly, despite clear mitochondrial dysfunction, the multisystem phenotypic appearance occurred in the absence of an overt oxidative stress imbalance, even under stressed conditions. Across models, basal and induced ROS levels, mitochondrial superoxide production, sensitivity to oxidative stressors and markers of lipid peroxidation or ferroptosis, were only modestly or incoherently affected. These findings, together with other experimental as well as clinical reports showing limited effects of antioxidant interventions^11,12,20,80,81^ argue against a primary role for oxidative damage in driving disease pathogenesis in the context of CI deficiency. Nonetheless, we cannot exclude that oxidative stress may instead play a more prominent role in other MD, in a mutation- or context-dependent manner. Identifying the genetic and metabolic pathways that buffer or exacerbate CI dysfunction^20,39,80,82,83^ will be essential for understanding phenotypic variability and developing targeted therapeutic strategies.

The shared pathological features observed across experimental systems, together with the rescue of key phenotypes by the structurally unrelated dietary compounds lutein and vitamin B12, suggest the existence of conserved metabolic programs that remain amenable to therapeutic modulation. While perturbations in NAD and lipid metabolism^84–88^ have emerged as hallmarks of mitochondrial dysfunction and may contribute to the beneficial effects of lutein and vitamin B12^89,90^, our findings point to one-carbon metabolism^75,91^ as a particularly attractive candidate linking CI dysfunction to downstream neuronal alterations. Vitamin B12 is a central regulator of one-carbon metabolism and choline homeostasis, pathways likely disrupted upon CI dysfunction through impairment of the NADH/NAD^+^ ratio and the methionine cycle. Consistent with this model, microbiota-derived vitamin B12 has been shown to suppress cholinergic hyperactivation by limiting choline availability at the synapse^92^ and we now found that choline supplementation rescued DGLA-induced sterility in *C. elegans*, while bacteria enriched in vitamin B12 rescue different *C. elegans* phenotypes. Moreover, we previously demonstrated that lutein restores cholinergic synaptic function in a *C. elegans* model of CI deficiency^12^ and we now find that both lutein and vitamin B12 rescue neurobehavioral phenotypes across disease models. Together, these observations suggest that these structurally distinct dietary compounds converge on interconnected one-carbon/choline-dependent pathways regulating cholinergic signaling and neuronal excitability, rather than acting through compound-specific mechanisms. This model is particularly compelling in light of the epileptic-like phenotypes observed in both brain organoids and *C. elegans*, suggesting that targeting downstream neuronal dysfunction may represent a feasible therapeutic strategy even in the presence of persistent mitochondrial defects. From a translational perspective, while ketogenic diets (with their limitations)^93–95^ have provided proof-of-principle that systemic metabolic modulation can improve disease outcomes, our study suggests that combination therapies targeting convergent metabolic pathways^96^ may represent an effective and clinically feasible approach.

Finally, the ability of dietary compounds to modulate neuronal phenotypes supports the contribution of systemic, non-cell-autonomous mechanisms to mitochondrial disease pathogenesis. Increasing evidence indicates that carotenoids, vitamins and microbiota-derived metabolites influence neuronal function through the gut-brain axis. Consistent with this concept, we observed diet-dependent phenotypic modulation in *C. elegans*, highlighting the importance of host-microbiota interactions and the value of multicellular models for capturing systemic mechanisms that cannot be reproduced in isolated cell systems. These findings further support a role for gene-environment interactions in the clinical heterogeneity among patients carrying similar mitochondrial mutations and highlight the need to understand how environmental and nutritional factors interact with mitochondrial dysfunction to develop personalized therapeutic strategies. In this context, the strength of this study lies in the integration of complementary experimental systems, ranging from cellular models to human iPSC-derived neuronal cultures and whole-animal models. This multisystemic pipeline captures complementary aspects of CI pathology, from mitochondrial dysfunction to neuronal circuit abnormalities and systemic phenotypes, while providing a robust platform for intervention studies. Although the molecular mechanisms linking CI dysfunction to one-carbon metabolism and neuronal signaling remain to be fully elucidated, our findings identify interconnected metabolic pathways as promising targets for future mechanistic investigation and therapeutic development.

In summary, the main advances of this study are: i) the establishment of isogenic patient-specific *NDUFS1* pipeline across complementary model systems, enabling identification of conserved disease endpoints in line with NAM development; ii) the identification of epileptic-like phenotypes as a novel translational readout; and iii) the discovery of lutein and vitamin B12 as dietary interventions supporting convergent metabolic mechanisms and cross-species phenotypic rescue.

## Supplementary Material

Supplementary Files

This is a list of supplementary files associated with this preprint. Click to download.

- Supplementarydata.docx

## Figures and Tables

**Figure 1:**
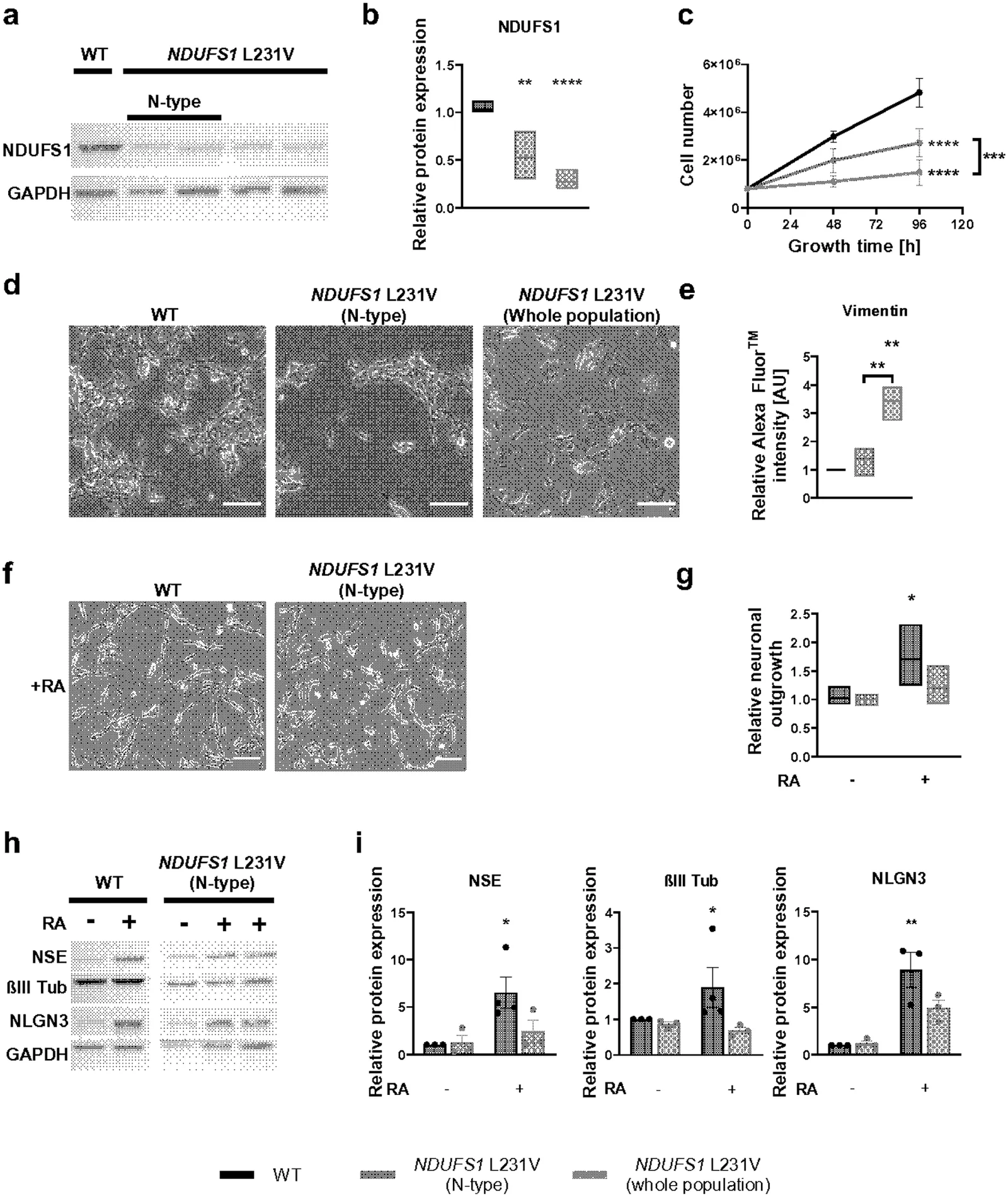
Mammalian SH-SY5Y cells carrying *NDUFS1* L231V mutation display decreased proliferation and differentiation capacity. **a)**Representative SDS PAGE and western blot analysis of NDUFS1 protein expression normalized to the housekeeping protein GAPDH in SH-SY5Y WT and *NDUFS1*L231V cells. **b)** Quantification of NDUFS1 protein levels normalized to GAPDH and relative to WT. Floating bars show mean with upper/lower bounds (N=4 independent replicates). Statistical analysis: Ordinary one-way ANOVA followed by Dunnett‘s multiple comparisons test. **p<0.01, ****p<0.0001. **c)** Proliferation curves of SH-SY5Y WT and *NDUFS1*L231V measured at 0, 48 and 96 h. Graph shows mean ± SD (N=3 independent replicates). **d)** Representative brightfield images of SH-SY5Y WT, *NDUFS1*L231V (whole population) and *NDUFS1* L231V (N-type enriched) cells. Images acquired at 20x magnification, scale bar=100 μm. **e)**Quantification of relative fluorescence intensity of immunostained vimentin in WT, *NUDFS1* L231V (N-type enriched) and *NDUFS1* L231V (whole population) cells. Bar graph shows mean ± SD (N=3 independent replicates). Statistical analysis: Ordinary one-way ANOVA followed by Tukey‘s multiple comparisons test. **p<0.01. **f)** Representative image of differentiated (RA +) WT and *NDUFS1* L231V (N-type enriched) cells. Images acquired at 20x magnification, scale bar=100 μm. **g)** Neuronal Outgrowth of undifferentiated and differentiated WT and *NDUFS1* L231V, quantified from brightfield and nuclear fluorescence images with ImageJ Plugin NeuriteTracer. Floating bars show mean with upper/lower bounds (N=3 independent replicates, n=4). Statistical analysis: Brown-Forsythe ANOVA followed by Dunnett’s T3 multiple comparisons test. *p<0.05 **h)** Representative SDS PAGE and western blot analysis of neuronal markers Neuron Specific Enolase (NSE), ßIII Tubulin (ßIII Tub) and Neuroligin3 (NLGN3) normalized to the expression of housekeeping protein GAPDH in WT and *NDUFS1* L231V cells. **i)**Quantification of NSE, ßIII Tubulin and NLGN3 relative to WT normalized to GAPDH. Bar graphs show the mean ± SD (N=4 independent replicates). Statistical analysis: Ordinary one-way ANOVA followed by Šídák’s multiple comparisons test. #p< 0.05. ##p< 0.01. Statistical analysis: One-way ANOVA followed by Šídák’s multiple comparisons. #p<0.05, ##p<0.01, ####p<0.0001. Asterisks (*) denote significant differences vs WT, hashtags (#) denote significant differences between treatment within the same cell line.

**Figure 2:**
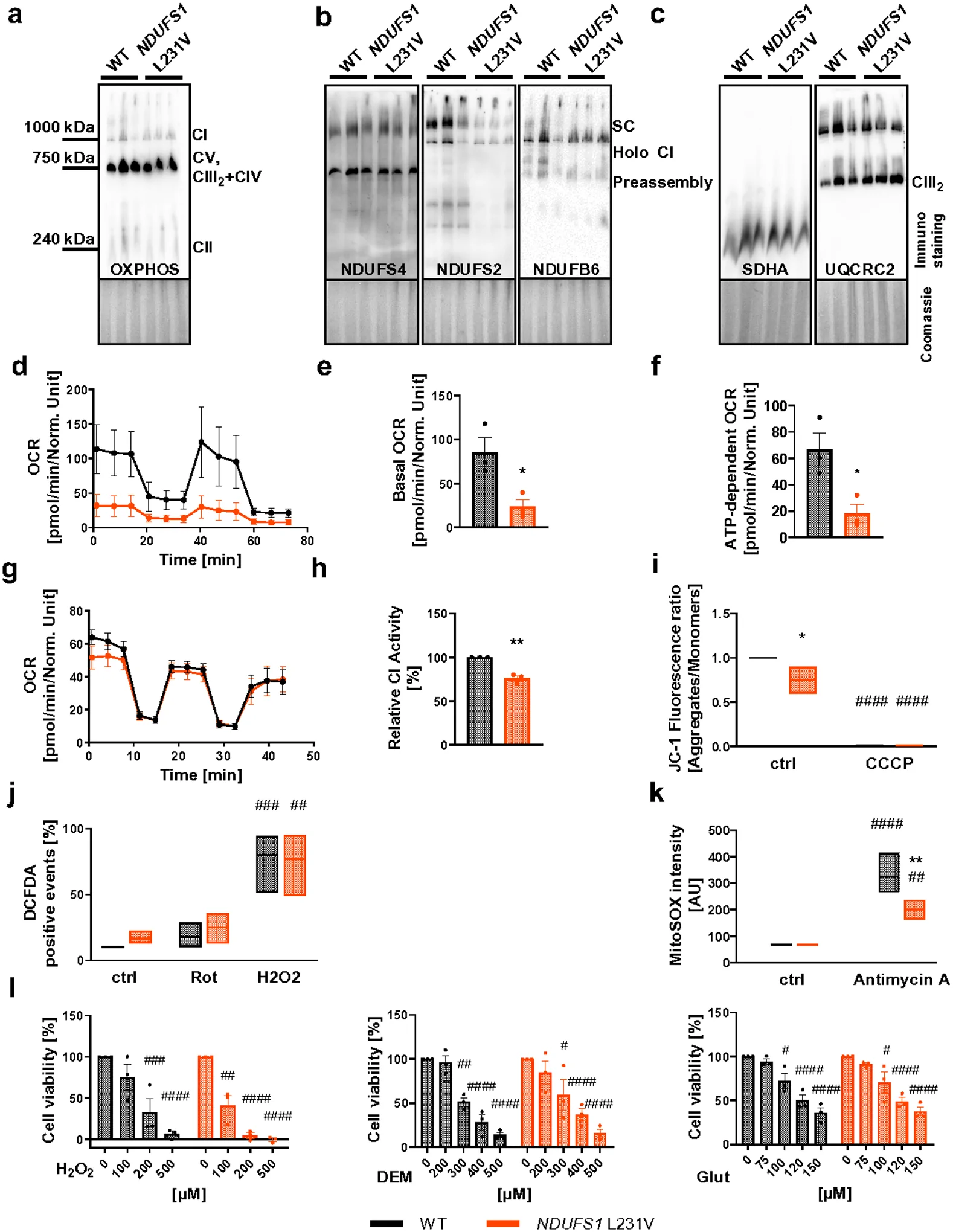
*NDUFS1* L231V mutated SH-SY5Y cells display mitochondrial alterations. **a)** Representative Blue Native (BN) PAGE and western blot analysis of mitochondrial ETC complexes and supercomplex (SC) formation in WT and *NDUFS1* L231V cells. Immunostained with OXPHOS antibody cocktail to visualize protein size and minor SC formation. **b)** BN-PAGE immunoblots of CI subunits (NDUFS4, NDUFS2 and NDUFB6) indicating presence of CI preassembly complexes, holoenzyme and SCs. **c)** BN-PAGE immunoblots of Complex II (SDHA) and Complex III (UQCRC2). Coomassie staining as loading control. Images display N=3 independent replicates. **d)** Seahorse XFe 96 Mito Stress Test respiration curves of WT and *NDUFS1* L231V showing mean ± SD of oxygen consumption rate (OCR) in response to indicated injections of oligomycin, FCCP and rotenone/antimycin A (N=3 independent replicates, n=15). **e)** Quantification of basal respiration and **f)** ATP production of WT and *NDUFS1* L231V showing mean ± SD (N=3 independent replicates. n=15 per condition). Statistical analysis: Welch’s t test, *p<0.05. **g)** Representative OCR respiration curve of WT and *NDUFS1* L231V in response to indicated injections of rotenone, succinate, antimycin A and TMPD/ascorbate, measured with Seahorse XFe 96 analyzer. **h)** Quantification of CI activity, showing mean ± SD, normalized on WT (N=3 independent replicates, n=15 per condition). Statistical test: unpaired t-test, **p<0.01. **i)** Mitochondrial membrane potential in WT and *NDUFS1* L231V in control (ctrl) and treated with CCCP, measured by JC-1 staining and FACS. Floating bars show means with upper/lower bounds (N=4 independent replicates). Statistical analysis: Two-way ANOVA followed by Šídák’s multiple comparisons test, *p<0.05, #p<0.0001). **j)** Cellular ROS in WT and *NDUFS1* L231V cells after rotenone and H_2_O_2_ treatment relative to WT ctrl. Floating bars show mean with upper/lower bounds (N=3 independent replicates). Statistical analysis: Two-way ANOVA followed by Tukey’s multiple comparisons test, ##p<0.01. ###p<0.001 **k)** Mitochondrial ROS in WT and *NDUFS1* L231V in ctrl and treated with antimycin A. Floating bars show MitoSOX intensity means with upper/lower bounds (N=3 independent replicates). Statistical analysis: Two-way ANOVA. **p<0.01, ##p<0.01. ####p<0.0001. **l)** Resazurin based relative cell viability in WT and *NDUFS1* L231V cells after treatment with H_2_O_2_, DEM or glutamate, showing mean ± SD (N=3 independent replicates, n=6). Statistical analysis: One-way ANOVA followed by Šídák’s multiple comparisons test. #p<0.05, ##p<0.01, ####p<0.0001. Asterisks (*) denote significant differences vs WT, hashtags (#) denote significant differences between treatment within the same cell line.

**Figure 3:**
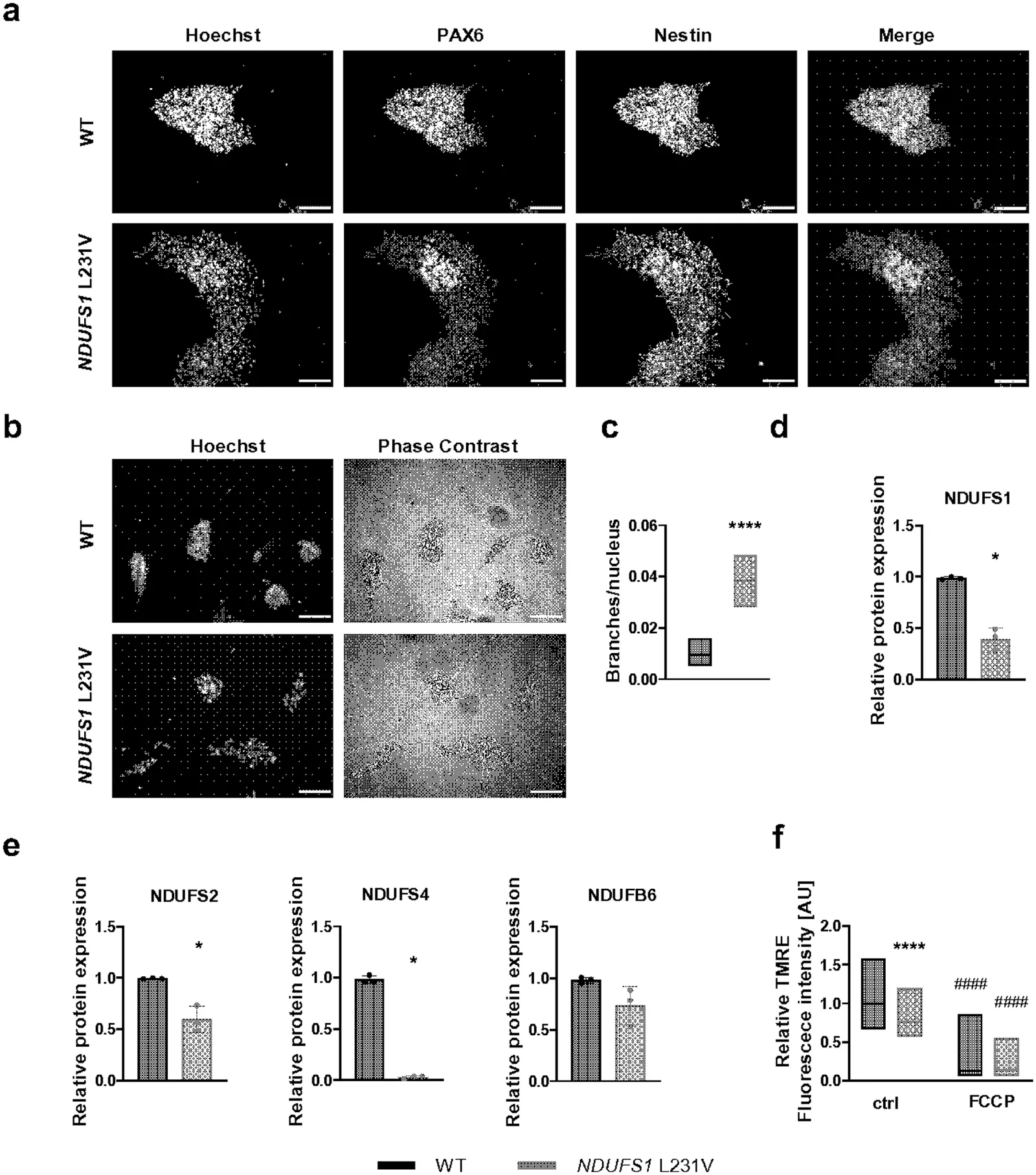
iPSCs-derived NPCs carrying *NDUFS1* L231V mutation display mitochondrial and neuronal defects. **a)** Representative fluorescence images characterizing WT and *NDUFS1* L231V NPCs. Images acquired at 20x magnification, scale bar = 100 μm. Hoechst = blue, PAX6 = green, Nestin = red. **b)** representative phase contrast and fluorescence images of early NPC branching. Nuclei were stained with Hoechst and counted with CellProfiler. Branches were identified and counted manually in phase contrast images. The branch count was divided by the respective nuclei count to obtain the Branches per Nucleus result. Scale bar = 100 μm. **c)** Quantification of early neuronal branching. Floating bars show mean and upper/lower bounds (N=3 independent replicates, n=5 per condition). Statistical analysis: Two-way ANOVA, ****p <0.0001. **d-e)** Quantification of NDUFS1, NDUFS2, NDUFS4 and NDUFB6 relative to WT normalized to GAPDH. Bar graphs show the mean ± SD (N=3 independent replicates, n=3 technical replicates). Statistical analysis: Welch’s t test, p<0.05. **f)** Quantification of mitochondrial membrane potential in untreated WT and *NDUFS1* L231V NPCs and treated with FCCP as positive control, floating bars show mean and upper/lower bounds of TMRM signal intensity in NPCs relative to WT ctrl. Statistical analysis: One-way ANOVA and Tukey’s multiple comparisons test ****p<0.0001, ####p<0.0001. Asterisks (*) denote significant differences vs WT, hashtags (#) denote significant differences between treatments within the cell line.

**Figure 4:**
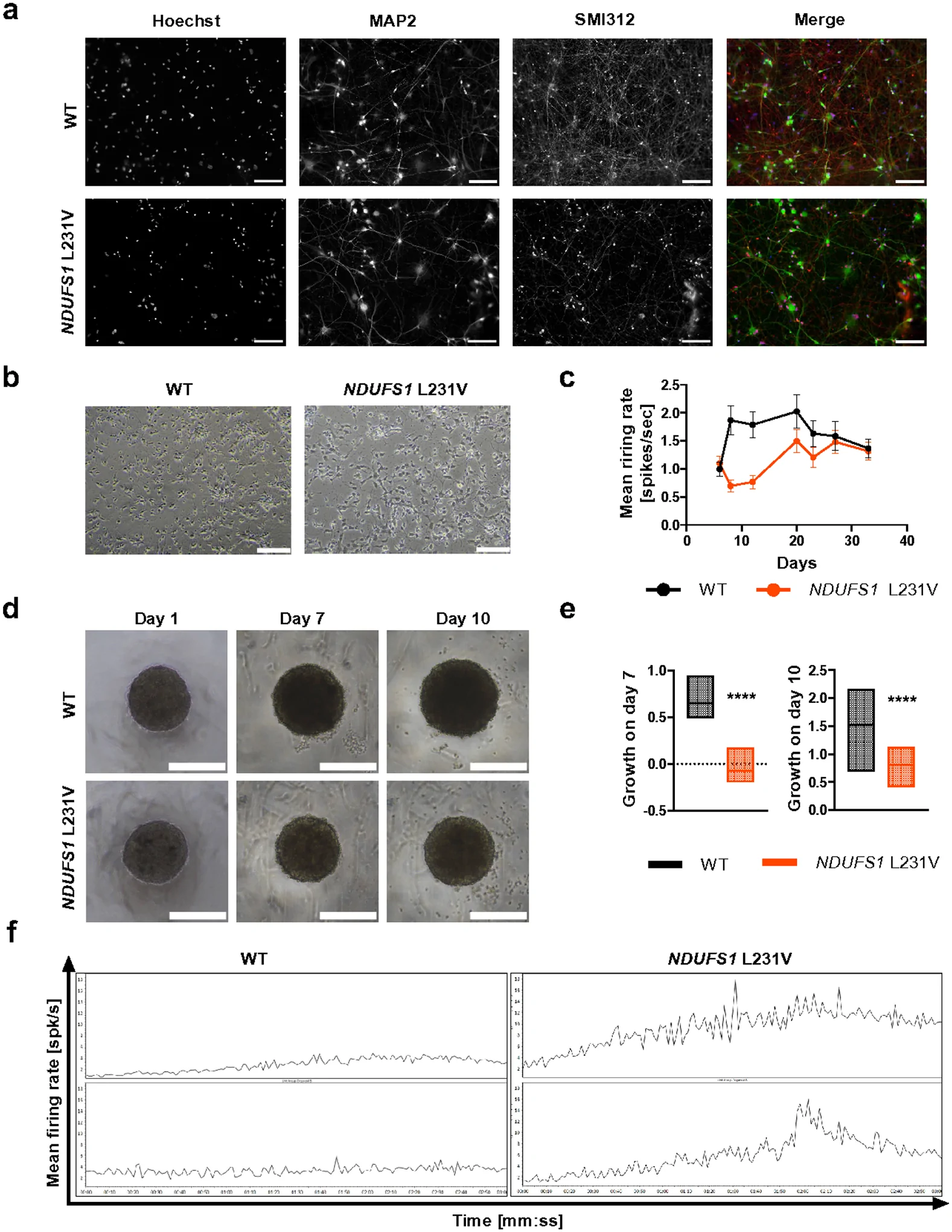
*NDUFS1* L231V NPCs-derived mDAN and MBOs display altered growth capacity and electrical activity. **a)** Representative fluorescence images characterizing WT and *NDUFS1* L231V mDANs. Merge colors: Hoechst=blue, MAP2=green, SMI312=red. Scale bar=100 μm. **b)** Representative brightfield images of mDAN neurite growth one day post-seeding. Scale bar=200 μm. **c)** High-density microelectrode array (HD-MEA) recording, measuring external electrical activity of mDAN cultures. Line graph depicting the mean firing rate and 95% confidence interval of the top 1% most actively spiking electrodes. **d)** Representative brightfield images of MBO growth. Scale bar=400 μm. **e)** Quantification of MBO growth from batch mean on day 1 and on day 10/12, floating bars show mean and upper/lower bounds of N=3 independent replicates (n=10). Statistical analysis: Two-way ANOVA, ****p <0.0001. **f)** Mean firing rate of two control and two mutant MBOs stimulated with 4-AP. Asterisks (*) denote significant differences vs WT.

**Figure 5:**
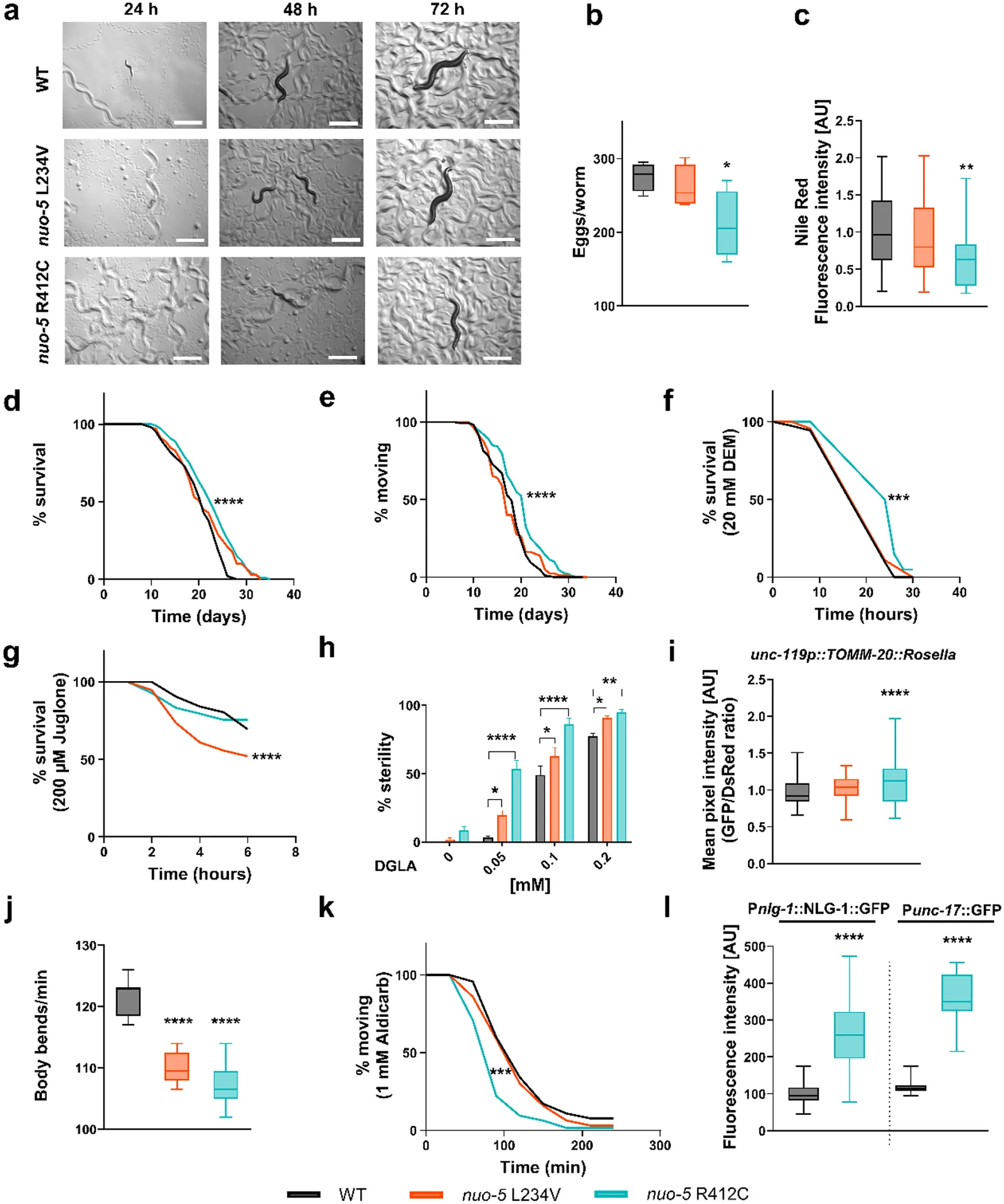
*C. elegans nuo-5* knock-in mutants present with phenotypic and neurobehavioral defects. **a)** Representative images of worms’ development after 24, 48 and 72 h after egg lay. Scale bar = 500 μm. Pictures were acquired with a Leica MZ10 F modular stereo microscope connected to a color digital camera. Three biologically independent experiments with similar results. WT and L234V reached adulthood and started laying eggs after 72 h but the R412C was still at a younger stage and not fertile yet at the same time point. **b)** Fertility (total brood size) N=2 independent replicates (n≥24 worms per condition), **c)** Quantification of the integrated intensity of Nile Red–stained lysosome-related organelles (LROs) in one-day adult worms. N=3 independent replicates (n≥36 worms per condition). Ordinary one-way ANOVA followed by Šídák’s multiple comparisons test N2 vs R412C *p*=0.0023 **d)** lifespan N=3 independent replicates (n≥60). Log-rank test WT vs R412C *p*=0.0001 and **e**) healthspan N=3 independent replicates (n≥60 worms per condition) of worms grown at 20 °C. Statistical analysis for lifespan: Log-rank test WT vs R412C *p*=0.0001 and for healthspan Log-rank test WT vs R412C *p*=0.0042. **f)** Survival curve on 20 mM DEM plates, N=3 independent replicates (n≥12), and **g)** survival curve on 200 μM juglone plates, N=3 independent replicates (n≥12), at first day adulthood and 20°C. Statistical analysis for DEM Log-rank test WT vs R412C *p*= 0.0032; for juglone Log-rank test WT vs R412C *p*=0.0069. **h)** DGLA-induced ferroptosis, N=4 n=50. Two-way-ANOVA followed by Dunnett’s multiple comparisons test. **i)** Neuronal mitophagy presented as ratio of GFP/DsRed mean pixel intensity in the head region of day 1. Nematodes express unc-119p::TOMM-20::Rosella in WT, *nuo-5*R412C and *nuo-5*L234V background. N=4, n=15–30 per condition. Scale bar=50 μm. **j)** Frequency of body bends in liquid media (“thrashing”) were quantified manually for one minute. N=3 independent replicates n≥45 worms per condition. **k)**Aldicarb-induced paralysis curves on plates containing 1 mM aldicarb. N=3 independent replicates n≥45 worms per condition. Log-rank test WT vs R412C p=0.00034. **l)** Quantification of GFP expression in P*nlg-1*::gfp and P*unc-17*::gfp transgenic strains. N=3 independent replicates n≥45 worms per condition. Bar graphs represent means ± SEM, Statistical analysis: One-way ANOVA followed by Fisher’s LSD test for multiple comparisons. *p<0.05, **p<0.01. Asterisks (*) denote significant differences vs WT. All assays were carried out on OP50 bacteria except in panel **k)** where animals were fed HT115 bacteria.

**Figure 6:**
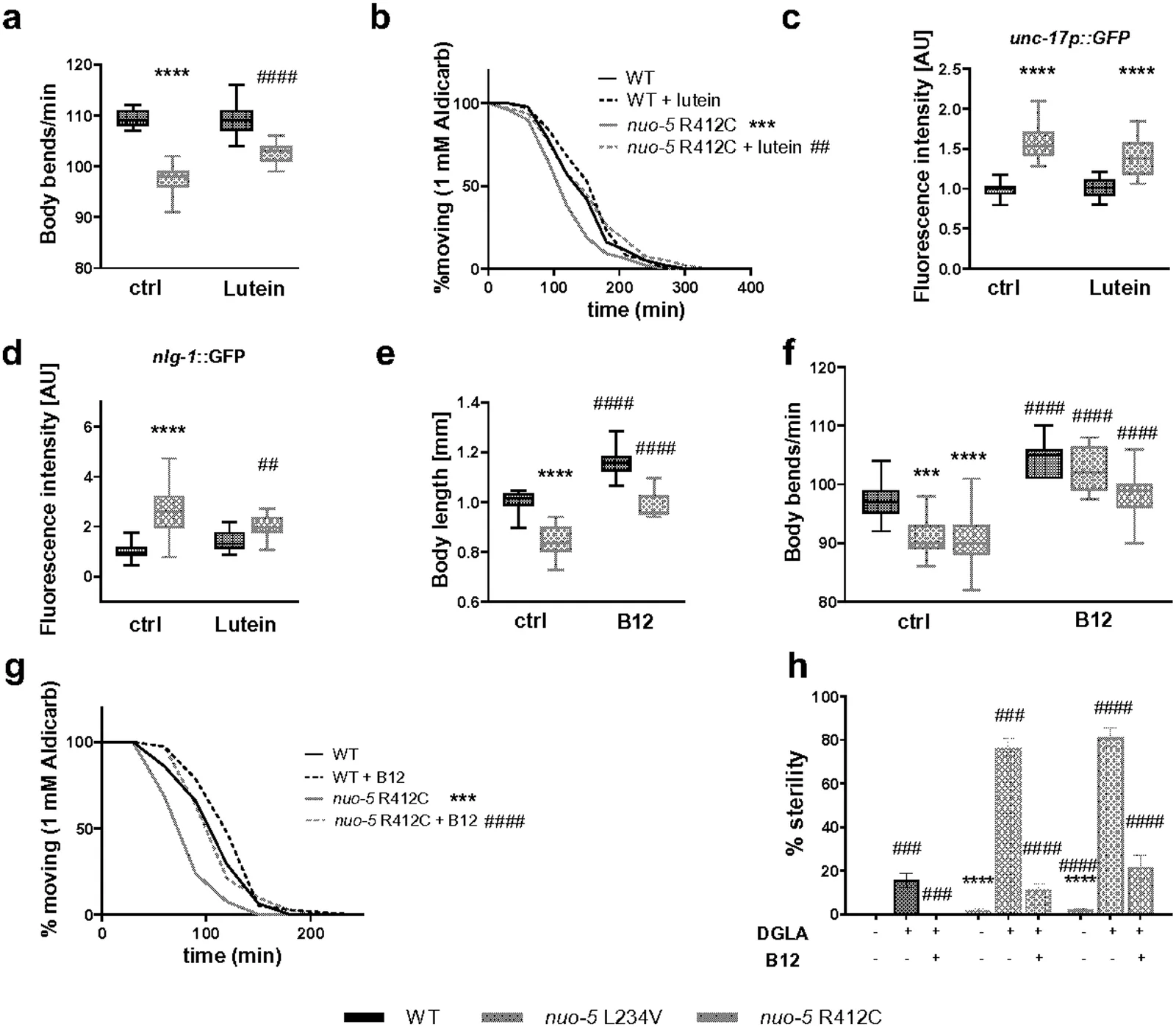
Lutein and vitamin B12 rescue neuronal deficits in the *C. elegans NDUFS1/nuo-5* mutants. **a)** Frequency of body bends in liquid media (“thrashing”) were quantified manually for one minute. N=3 independent replicates n≥45. **b)** Aldicarb-induced paralysis curves on plates containing 1 mM aldicarb. Log-rank test: WT vs R412C p =0.0002, N=3 n ≥ 75 worms per condition. **c)** Quantification of GFP expression in P*nlg-1::*gfp transgenic strain. N=3 n ≥ 30 worms per condition. **d)**Quantification of GFP expression in P*unc-17*::gfp transgenic strain. N=3 n ≥ 30 worms per condition. One-way ANOVA **e)** worms’ length from eggs and from L4 larvae. **f)** Motility in liquid, manual assay. N=3, n =45 worms per condition. **g)**Aldicarb sensitivity Log-Rank test WT vs R412C *p*= 0.0397, R412C vs R412C + lutein *p*= 0.0136, **h)** DGLA-induced ferroptosis, N=4, n=50.1 μM lutein in **a**, **b**, **c** and **d**, 100 nM B12 in **e**, **f**, and **g**, in **h**1 nM B12. Bar graphs represent means ± SEM, Statistical analysis: One-way ANOVA followed by Fisher’s LSD test for multiple comparisons. *p<0.05, **p<0.01. Asterisks (*) denote significant differences vs WT, hashtags (#) denote significant differences between treatments within the same strain. All panels on OP50 bacteria.

**Figure 7:**
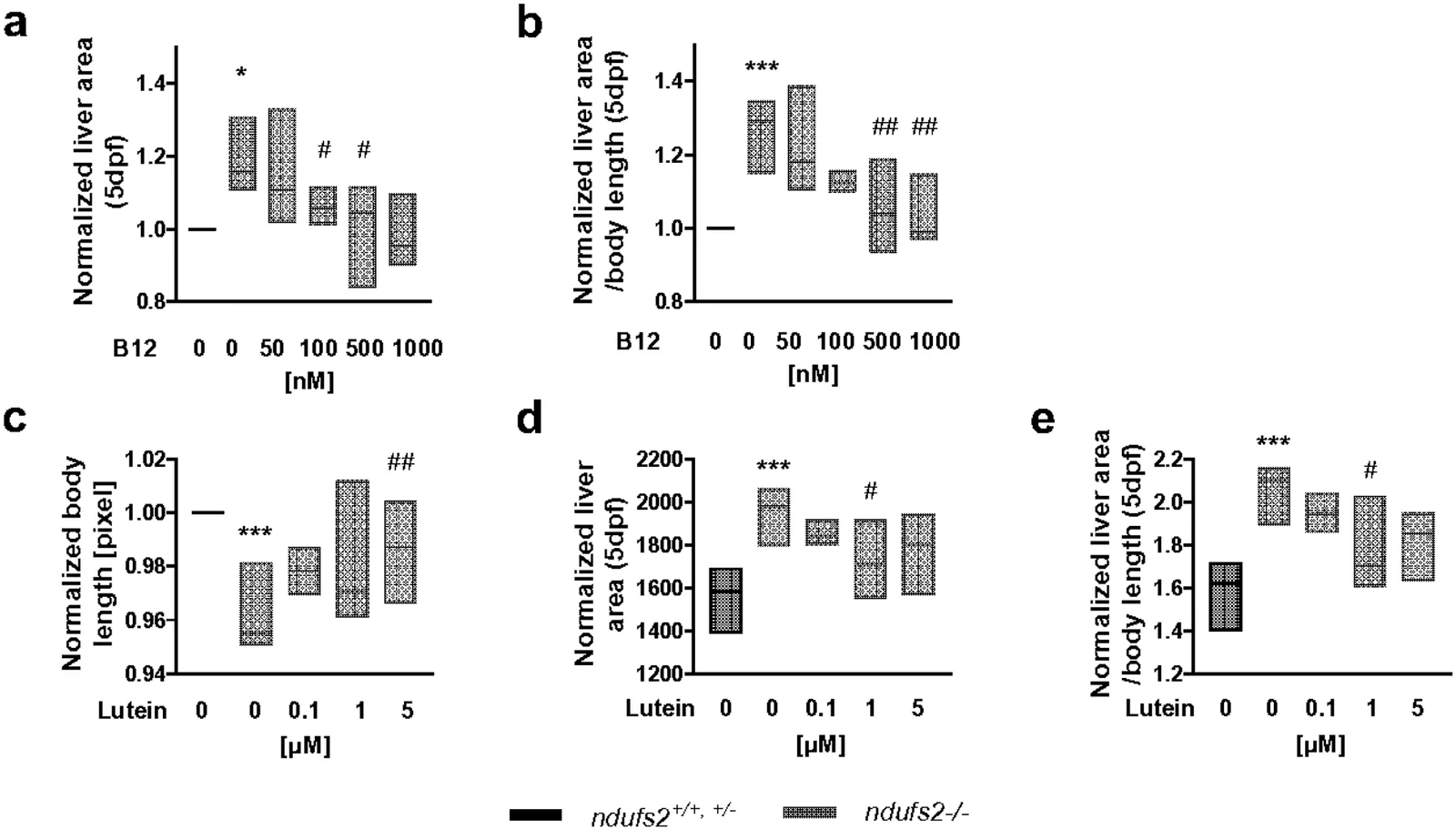
Lutein and vitamin B12 rescue neurometabolic deficits in zebrafish CI mutants. **a)** Liver Area and **b)** liver area normalized on body length in zebrafish larvae 5 days post fertilization (dpf) treated with different concentrations of B12. Floating bars show mean and upper/lower bounds of N=5 (except for 100 and 1000 nM where N=3), n≥30. **c)** Body length**, d)** Liver Area and **e)** liver area normalized on body length in zebrafish larvae 5 dpf) treated with different concentrations of lutein. Floating bars show mean and upper/lower bounds of N=5 (except for 0.1 μM lutein where N=3), n≥30. Statistical analysis: One-way ANOVA followed by Šídák’s multiple comparisons test. *p<0.05, ***p<0.001, #p<0.05, ##p<0.01. Asterisks (*) denote significant differences vs control, hashtags (#) denote significant differences between doses within the same strain.
